# Extensive structural rearrangement of intraflagellar transport trains underpins bidirectional cargo transport

**DOI:** 10.1016/j.cell.2024.06.041

**Published:** 2024-08-22

**Authors:** Samuel E. Lacey, Andrea Graziadei, Gaia Pigino

**Affiliations:** 1 Human Technopole, Milan 20157, Italy

**Keywords:** cilia, intraflagellar transport, cryoelectron tomography, retrograde transport, molecular structure

## Abstract

Bidirectional transport in cilia is carried out by polymers of the IFTA and IFTB protein complexes, called anterograde and retrograde intraflagellar transport (IFT) trains. Anterograde trains deliver cargoes from the cell to the cilium tip, then convert into retrograde trains for cargo export. We set out to understand how the IFT complexes can perform these two directly opposing roles before and after conversion. We use cryoelectron tomography and *in situ* cross-linking mass spectrometry to determine the structure of retrograde IFT trains and compare it with the known structure of anterograde trains. The retrograde train is a 2-fold symmetric polymer organized around a central thread of IFTA complexes. We conclude that anterograde-to-retrograde remodeling involves global rearrangements of the IFTA/B complexes and requires complete disassembly of the anterograde train. Finally, we describe how conformational changes to cargo-binding sites facilitate unidirectional cargo transport in a bidirectional system.

## Highlights

• Cryo-ET structure of retrograde IFT trains
• IFTA forms the core and makes symmetrical polymeric interfaces
• IFT train remodeling at the tip involves complete disassembly of anterograde train
• Remodeling generates unique cargo-binding surfaces

## Graphical abstract

**Figure:**
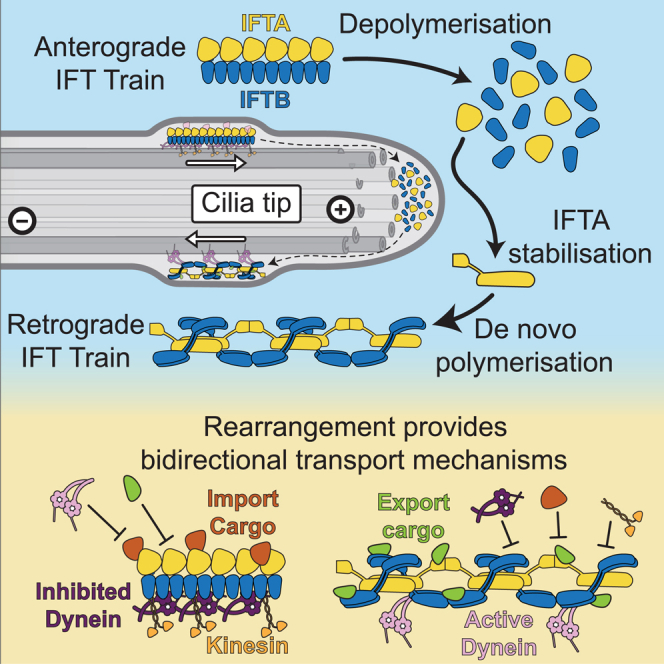

## Teaser

Visualizing retrograde IFT trains by *in situ* cryoelectron tomography and structural proteomics reveals how trains are remodeled at the cilium tip and provides insight into the regulation of selective cargo transport.

## Introduction

Primary and motile cilia are microtubule-based organelles that are separated from the bulk cytoplasm by a diffusion barrier called the transition zone.^1^ This enables the creation of a specialized ciliary proteome and concentrates many copies of structural proteins that self-organize into the ciliary axoneme. Furthermore, the regulated enrichment or removal of functional proteins, such as receptors, is a key step in the activation of many signaling cascades that operate in primary cilia.^2^ The cell controls which proteins can pass through the transition zone with the intraflagellar transport (IFT) system,^1^^,^^3^^,^^4^ which involves the bidirectional active transport of cargoes into and out of the cilium. IFT is essential for proper cilia assembly, maintenance, and function, and mutations that affect IFT components are a leading cause of ciliopathies in humans.^2^

IFT is powered by microtubule motors and is orchestrated by the IFTA and IFTB protein complexes, comprising of 6 and 16 subunits, respectively.^5^ Together, IFTA and IFTB polymerize into IFT trains that act as adaptors between the cargoes and the motors. Kinesin-2 motors power anterograde trains, which polymerize at the base of the cilium (the basal body),^6^ import cargoes from the cytoplasm, and deliver them to the cilium tip.^1^^,^^4^^,^^7^^,^^8^ Here, the anterograde trains undergo a poorly understood remodeling process and convert into retrograde trains.^9^ The retrograde train can now recruit active dynein-2 motors and move signaling effectors and proteins destined for recycling or degradation back to the cell body. Therefore, IFT has completely segregated its plus- and minus-end-directed phases of transport. This is unlike other long-range bidirectional transport systems, such as vesicle transport in neurons, which display frequent pauses and switches in direction.^10^^,^^11^

As such, a key step in the IFT cycle is the rapid conversion from anterograde trains to retrograde trains at the tip. However, the mechanism of remodeling is unknown. One model suggests that anterograde trains are internally remodeled at the tip.^12^ Spring-like structural domains in both the IFTA and IFTB complexes could generate the conformational changes underlying the remodeling process.^13^^,^^14^ In this model, the lateral interactions between adjacent IFT complexes would broadly be retained from the anterograde-to-retrograde trains. Alternatively, it has been proposed that the anterograde trains disassemble at the tip, with the individual IFTA and IFTB complexes repolymerizing into an entirely novel polymer.^15^ However, visualization of such a dynamic process at a high enough resolution has proven to be extremely challenging, and the mechanism of IFT train conversion is still unknown.

Another fundamental question in the IFT cycle is how anterograde and retrograde trains bind to different motors and cargoes. Cargo selectivity ensures that only specific ciliary components can be accumulated or removed from the cilium, thus underpinning its entire organization. Unique in the IFT system is dynein-2, which acts differentially as a cargo and motor on anterograde and retrograde trains, respectively.^16^^,^^17^ First, it is carried as an autoinhibited cargo on the anterograde train before re-binding and powering the movement of the retrograde train in the active state. Most cargoes tend to move unidirectionally, however. Live-cell imaging has shown that cargoes such as RSP3,^18^ DRC2/4,^19^^,^^20^ and tubulin^21^^,^^22^ are almost exclusively carried by anterograde IFT trains. Conversely, various ciliary membrane proteins, including PTCH1 and GPR161 from the hedgehog signaling pathway, are specifically removed from cilia upon signaling activation.^23^ Overall, direction-specific interactions with both motors and cargo must overcome the fact that anterograde and retrograde trains are made with a single set of IFTA/B complexes.

**Figure 1:**
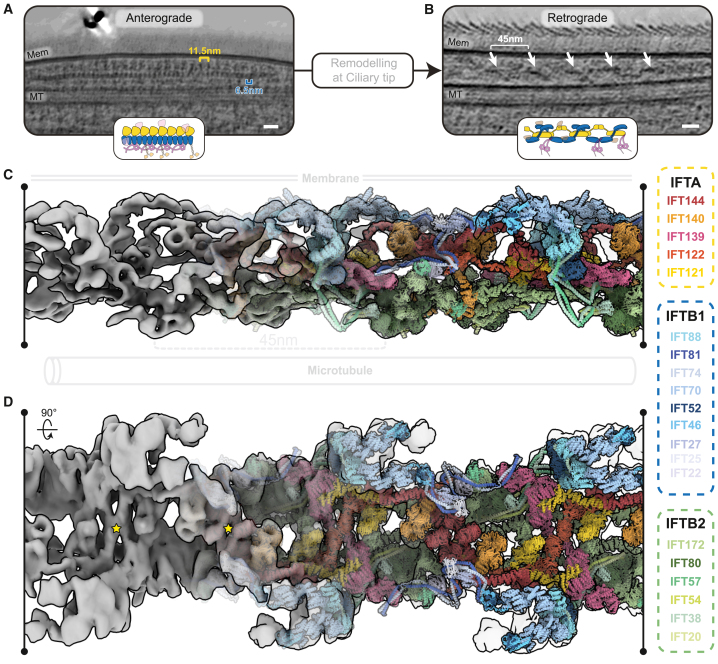
A molecular model of the retrograde IFT train (A) Slice through denoised cryoelectron tomogram of a wild-type *C. reinhartii* cilium, showing an anterograde IFT train sitting between the membrane (Mem) and microtubule (MT) doublet. Cartoon inset: IFTA, yellow; IFTB, blue; autoinhibited dynein-2, purple; kinesin, orange; cargo, pink. Scale bars, 25 nm. (B) Slice through denoised cryoelectron tomogram of a wild-type *C. reinhartii* cilium, showing a retrograde IFT train sitting between the membrane (Mem) and microtubule (MT) doublet. White arrows indicate equivalent structures in the repeat, with a spacing of ∼45 nm. Cartoon inset as in (A), but with different cargoes, brown. Scale bars, 25 nm. (C) A side view of the retrograde train density (microtubule below, membrane above), with the molecular model docked. The density becomes increasingly transparent from left to right. (D) Top view of (C), as if looking down from the membrane. Yellow stars indicate the center of rotation for the two C2 symmetry axes. See also Figure S1.

Recently, we presented *in situ* cryoelectron tomography (cryo-ET) structures of anterograde IFT trains, providing snapshots of the IFTA and IFTB complexes within the IFT cycle.^13^^,^^16^ The anterograde train consists of a dense IFTB polymer facing the microtubule and a separate but tightly associated IFTA polymer sitting on top and facing the membrane (Figure 1A). Several single-particle cryo-EM structures of purified IFTA complexes highlight the flexibility of this complex in solution prior to polymerization.^24^^,^^25^^,^^26^^,^^27^ However, our understanding of the IFT cycle is currently incomplete since we are lacking any structural information of retrograde IFT trains.

To understand how anterograde and retrograde IFT trains perform their opposing functions in bidirectional ciliary transport, we used cryo-ET of *Chlamydomonas reinhardtii* cilia, subtomogram averaging, and *in situ* cross-linking mass spectrometry (XL-MS) to obtain a structural model of retrograde IFT trains. This showed that retrograde IFT trains are non-polar, symmetrical polymers, with a central thread of IFTA complexes. We compare the structures of anterograde and retrograde IFT trains and show that the large-scale rearrangements between the two can only be achieved following the complete disassembly of anterograde trains into individual IFTA and IFTB complexes. We describe how a key conformational change in IFTA is required for the polymerization of retrograde trains. Finally, we explore how the anterograde and retrograde trains can differentially bind to cargoes based on their structural differences. Thus, in this study, we explain how IFT trains remodel at the tip and provide mechanisms for direction-specific cargo binding and transport in cilia.

## Results

### Retrograde trains are highly flexible polymers

**Figure S1:**
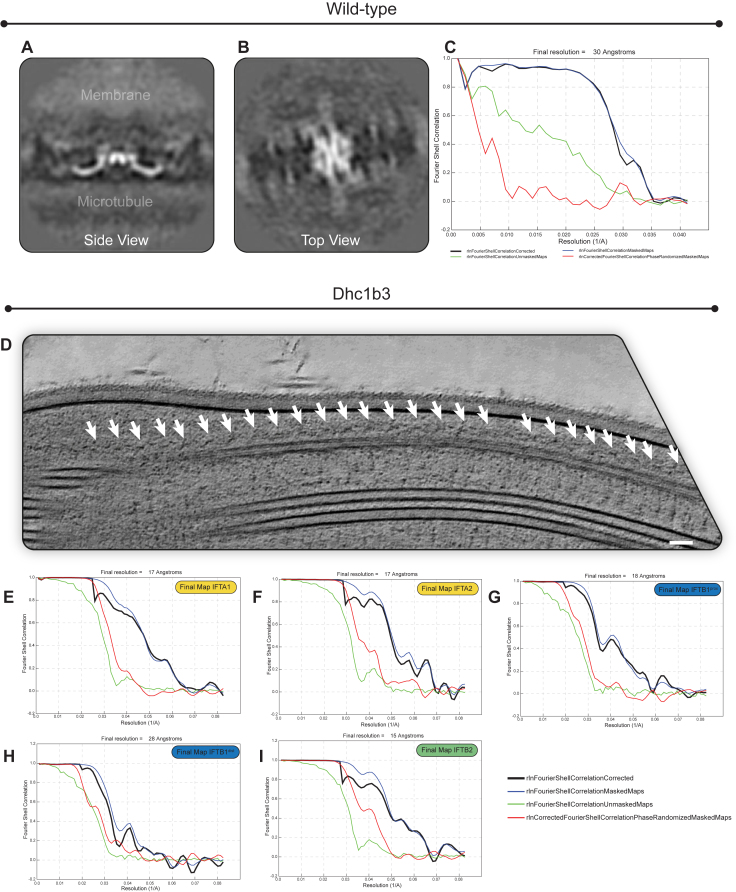
Cryo-ET and subtomogram averaging of wild-type and *Dhc1b-3* retrograde trains, related to Figure 1 (A) Side view slice through the wild-type retrograde train average. (B) Top view of (A), as if viewing down from the membrane. (C) Fourier shell correlation (FSC) plot for the 30.3 Å wild-type average. (D) A slice through a Dhc1b-3 tomogram, indicating long rafts of repeating retrograde train structures (white arrows). Scale bar corresponds to 25 nm. (E) Fourier shell correlation (FSC) plot for the 17 Å Dhc1b-3 IFTA1 average. (F) Fourier shell correlation (FSC) plot for the 17 Å Dhc1b-3 IFTA2 average. (G) Fourier shell correlation (FSC) plot for the 18 Å Dhc1b-3 IFTB1^prox^ average. (H) Fourier shell correlation (FSC) plot for the 28 Å Dhc1b-3 IFTB1^dist^ average. (I) Fourier shell correlation (FSC) plot for the 15 Å Dhc1b-3 IFTB2 average.

To solve the structure of retrograde IFT trains, we first identified retrograde trains in our previous dataset of wild-type *C. reinhardtii* cilia.^13^ Consistent with images from plastic-embedded transmission electron microscopy (TEM) images,^8^ anterograde and retrograde trains were easily distinguishable in these tomograms. Anterograde trains are highly electron dense and consist of two rigid stacked (IFTA/IFTB) polymers, each with distinct repeat distances (11.5/6.5 nm)^16^ (Figure 1A). By contrast, retrograde trains are much less electron dense and contain only one repeating structure (Figure 1B). With a repeat distance of ∼45 nm, the retrograde train is much more elongated and contains greater flexibility between adjacent repeats. We adapted our subtomogram averaging workflow to deal with the increased flexibility (see Methods S1) and obtained a 30.3 Å resolution reconstruction of the retrograde train (Figures S1A–S1C).

We concluded that the low resolution of this reconstruction was primarily caused by a low particle number. In 600 wild-type tomograms, we only identified 95 retrograde trains, compared with 741 anterograde trains. This is at odds with measurements from light microscopy suggesting that there should be roughly the same number of anterograde and retrograde trains present in the cilium at any one time.^28^^,^^29^ We suspected that the discrepancy is due to the retrograde trains being more delicate and thus more sensitive to disruption by the external forces that act upon the cilia during plunge freezing.

To obtain more particles, we sought to stabilize the retrograde trains *in situ*. TEM of *C. reinhardtii* with mutations in the retrograde dynein motor machinery previously revealed the formation of large “rafts” of non-motile IFT complexes in what resembled the retrograde conformation.^7^^,^^30^ We wanted to see if these trains are more stable targets for *in situ* structural characterization.

We focused our efforts on the *C. reinhardtii dhc1b-3* temperature sensitive mutant since it retains full-length cilia suitable for imaging when moved to the restrictive temperature.^31^ The mutation to the dynein heavy chain prevents the motor from entering the cilia, thus impairing the retrieval of retrograde trains back to the cell body.^31^ In cryoelectron tomograms of *dhc1b-3* cells at the restrictive temperature, there are large rafts of IFT trains that closely resemble the retrograde trains in our wild-type tomograms (Figure S1D). However, they are now much longer and regular. We collected a new dataset of 736 tomograms and manually identified 1,255 retrograde trains, containing 10,455 repeating units in total (Table S1).

Most particles that we could identify in our tomograms were side views (Figure S1D), resulting in partially anisotropic reconstructions. We performed masked refinements of four domains that displayed considerable flexibility relative to each other (see Methods S1). These produced maps ranging in resolution from 15 to 28 Å (Figures S1E–S1I), which we combined into a single composite map. The density from the *dhc1b-3* mutant matched the wild-type retrograde train (Figures S1A and S1B), thus confirming that we are observing IFT complexes in the retrograde conformation.

We next started to build up a molecular model of the proteins in our densities (see Methods S1). The IFTB complex consists of two biochemically characterized subcomplexes, IFTB1 and IFTB2. Structurally, these form two lobes separated by a flexible coiled coil.^13^^,^^32^ Similarly, the IFTA complex contains two lobes separated by a flexible domain, referred to as IFTA1 and IFTA2.^24^^,^^25^ We split IFTA/B into these four subcomplexes and found positions where each could be confidently docked into the density. Following additional AlphaFold modeling and molecular dynamics flexible fitting, we obtained our final model of the complete retrograde IFT train (Figures 1C and 1D; Video S1).

Video S1. A 360° rotation of the retrograde train composite map, followed by the same rotation with the refined model docked, related to Figure 1

### Retrograde IFT trains have 2-fold rotational symmetry

Unexpectedly, our structure shows that the retrograde train possesses 2-fold rotational symmetry perpendicular to the microtubule track (Figure 1D). This means retrograde trains do not possess directional polarity, despite being involved in unidirectional transport. It follows that if dynein-2 attaches to a structured subunit on the side of the train, only half of the motors will be facing the direction of transport. In the *dhc1b-3* cells, dynein is scarce in the cilium,^31^ meaning that the average will contain no density for dynein. However, in the wild-type average, there is no extra density that would indicate the dynein binding site (Figures S1A and S1B), suggesting that dynein binds to flexible or disordered domains of the retrograde train. This would allow dynein motors to always orient themselves correctly on the microtubule, despite the IFT complex they are attached to facing the “wrong” way. However, further work is needed to determine how dynein interacts with the retrograde train.

### IFTA is central to the retrograde train

**Figure 2:**
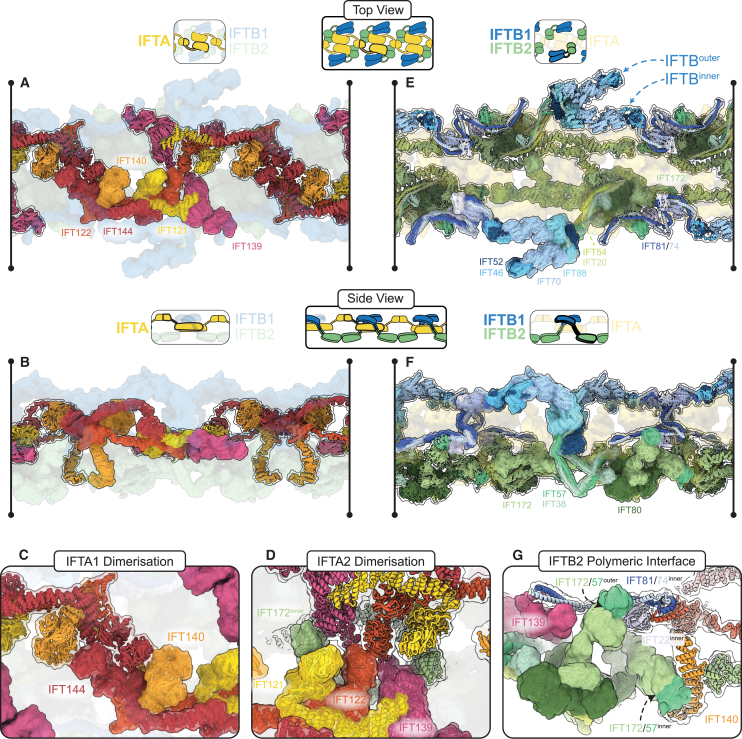
IFTA forms the core of the retrograde train and establishes C2 symmetry (A) Top view of the retrograde train as if looking down from the membrane, highlighting the IFTA complexes. IFTA is shown in ribbon representation, with a single central complex highlighted with a semi-opaque surface. IFTB is shown as a semi-transparent surface. (B) Side view of (A), with the membrane on top and the microtubule below. (C) Close up of the IFTA1 dimerization interface. Two copies of IFT144/140 are shown, with adjacent IFTA complexes depicted with different surface opacity. (D) Close up of the IFTA2 dimerization interface. Two copies of IFT144/140/139 are shown, with adjacent IFTA complexes depicted with different surface opacity. IFT172^inner^ is also present. (E) Top view of the retrograde IFT model as if looking down from the membrane, highlighting IFTB complexes at the periphery of the train. IFTB complexes are shown in ribbon representation, with one pair of IFTB complexes from one repeating unit also depicted with a semi-opaque surface. IFTA complexes are shown as a transparent surface. (F) Side view of (E), with the membrane on top and microtubule below. (G) Close up of the IFTB2 polymeric interface, with one repeating unit shown with semi-opaque surfaces and the other transparent. The two copies of IFT172/57 from IFTB2 adopt different conformations to form two separate interactions with the adjacent repeat. See also Figure S2.

IFTA complexes form a continuous central thread along the length of the retrograde train (Figures 2A and 2B). The IFTA complexes polymerize through two separate homodimeric interfaces between different neighboring complexes. First, the WD domains of two pairs of IFTA1 subcomplexes (IFT144/140-WD) wrap around each other as interlocking “hooks” (Figure 2C). In the second, two antiparallel IFTA2 subcomplexes stack against each other through IFT139 and the IFT122 and IFT121 WD domains (Figure 2D). Together, these two previously uncharacterized interactions form the basis for retrograde train polymerization and are responsible for the establishment of 2-fold symmetry. This arrangement is perhaps surprising since it positions the IFTA complex away from the membrane, where many of its interaction partners are located.

**Figure S2:**
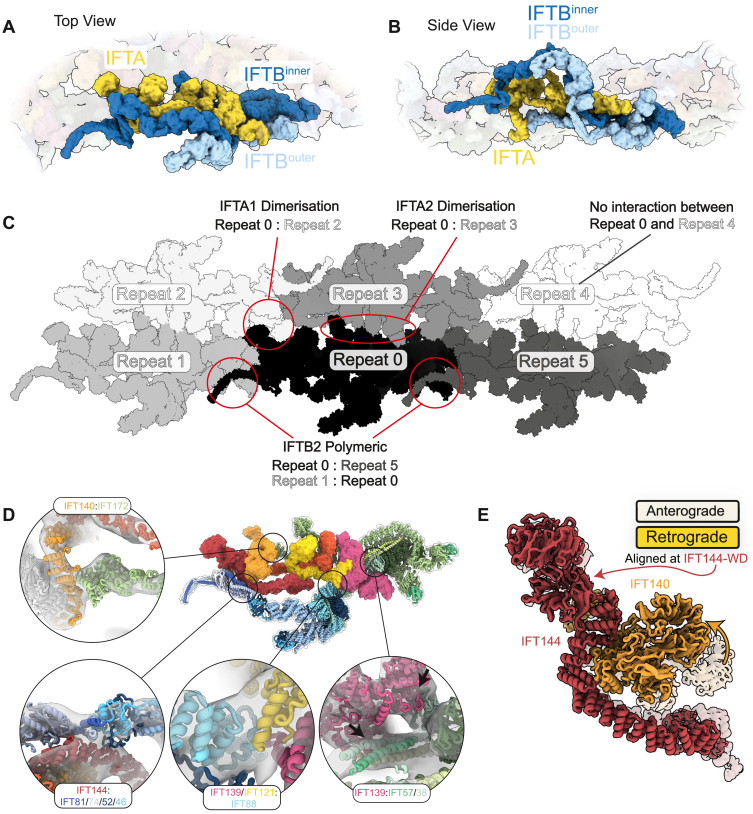
Structural analyses of the retrograde train, related to Figures 2, 3, and 4 (A) Top view of the retrograde train as if looking down from the membrane. One repeating unit is shown with an opaque surface. The IFTA complex is colored yellow. IFTB^inner^ and IFTB^outer^ are differentiated by different shades of blue. (B) Side view of (A), as if the membrane is above and the microtubule is below. (C) One central repeating unit (black, corresponding to one IFTA complex and the two IFTB complexes attached to it) makes contact with four surrounding repeating units (colored with different shades of gray). Only repeating unit 4 does not interact with the central repeating unit 0. (D) Close-up views of each other interactions between IFTA and IFTB in one repeating unit of the retrograde train, shown docked into the experimental density. (E) Comparison between IFTA1 (IFT144, red, IFT140, orange) in the anterograde (transparent) and retrograde (opaque) conformations. The two models are aligned to the IFT144 WD domain. Movement of the IFT144 TPR domain associated with bridge formation has moved the IFT140 WD domains closer to the IFT144 WD domains.

Conversely, IFTB forms the exterior “wings” of the retrograde train (Figures 2E and 2F). The IFTB1 subcomplex forms the membrane-proximal roof of the train, whereas the IFTB2 subcomplex forms the microtubule-proximal floor. There are two IFTB complexes (IFTB^inner^ and IFTB^outer^) wrapped around each IFTA complex (Figures 2E, 2F, S2A, and S2B), each adopting unique conformations. Thus, the basic repeating unit of the retrograde train consists of one IFTA complex and two IFTB complexes. This neatly matches the roughly 2:1 ratio of IFTB to IFTA observed in anterograde trains and therefore explains how the retrograde train transports all the IFT complexes at the tip to the base.

### The IFTB complex contributes to polymeric interfaces

At first glance, the two IFTB complexes may be seen as peripherally attached to the retrograde train, but both copies are integrally involved in polymeric interfaces. The IFT172^inner^ C terminus contributes to the IFTA2 dimeric interface (Figure 2D), bridging the two halves together. Furthermore, IFTB2^inner^ and IFTB2^outer^ form a separate polymeric interface to a third repeating unit (Figures 2G and S2C). IFT172/57^outer^ contacts IFT81/74/22^inner^ from an adjacent repeat, while IFT172/57^inner^ contacts the C terminus of the adjacent IFT122. Therefore, while retrograde train polymerization is primarily established by IFTA, it is completed by IFTB.

A final connection between adjacent repeats is provided by the continuation of the IFT81/74^inner^ coiled coils (Figures 2G and S2C). Beyond the interaction with IFT172/57^inner^, they go on to contact IFT139 from IFTA2 from the next repeating unit. This is the same interaction we identified between IFTA and IFTB in the anterograde train. Taken together, these polymeric interactions mean that each repeating unit contacts four other repeats, making the retrograde train a highly interconnected polymer.

### IFTA and IFTB are rearranged in the retrograde train

**Figure 3:**
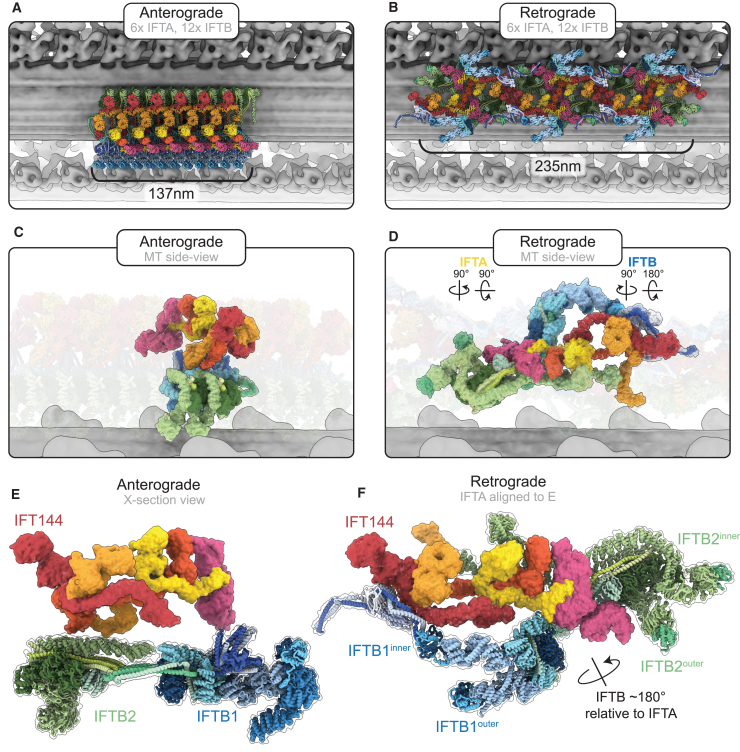
IFTA and IFTB are rearranged in retrograde trains (A) Top view of the anterograde train, with a microtubule doublet beneath in gray. (B) Top view of the retrograde train, with a microtubule doublet beneath in gray. The same number of IFTA/B complexes are shown as in the anterograde train in (A), but the length of the train is ∼70% longer. (C) Side view of the anterograde train, with the microtubule below and the membrane above. One IFTA complex and two IFTB complexes (equivalent to a repeating unit in the retrograde train) are highlighted. (D) Side view of the retrograde train, with one repeating unit highlighted. Compared with (C), the IFT complexes have undergone large movements relative to the microtubule and each other. (E) Cross-section view of the anterograde train, as if looking down the microtubule. IFTA sits on top of a platform of IFTB. (F) One repeating unit of the retrograde train, aligned so that the IFTA complex is oriented the same as in (E). The IFTB complexes have rotated 180° relative to the IFTA and are now wrapped around the IFTA. See also Figure S2.

Equipped with our new model of the retrograde train, we could start assessing the differences between anterograde and retrograde trains. Most noticeably, a retrograde train is ∼70% longer than an anterograde train containing the same number of IFT complexes (Figures 3A and 3B).

The overall architecture of each of the IFT subcomplexes (IFTA1/A2, IFTB1/B2) is broadly consistent between the anterograde and retrograde trains. However, they all undergo significant rearrangements with regards to each other and their environment. In the anterograde train, the elongated IFTA and IFTB complexes are both perpendicular to the microtubule, helping create the compact stacked structure (Figure 3C). In the retrograde train, IFTA and IFTB are both now parallel with the microtubule, explaining the expansion of the retrograde repeat distance (Figure 3D). Both complexes undergo further rotations relative to each other. The IFTA complex is rotated 90° around its own long axis, leaving it flat with the microtubule. This moves regions that formed the membrane-proximal roof of the anterograde train to the core of the retrograde train. Furthermore, it means one side of IFTA is now facing the microtubule and is thus inaccessible to the membrane. As such, the membrane-proximal “carriages” proposed for cargo binding in the anterograde train^33^ are absent in the retrograde train. In IFTB, there is a further 180° rotation relative to the microtubule. This flips the complex around, such that the regions that were facing the microtubule in the anterograde train are now membrane proximal. Together, these changes between the anterograde train and retrograde train completely alter how IFTA and IFTB are able to interact with their surroundings.

Importantly, these changes mean IFTA and IFTB are flipped relative to each other in the retrograde train. This is highlighted when we align IFTA from our anterograde and retrograde models and look at the position of IFTB proteins (Figures 3E and 3F). In the anterograde train, IFTB2 is directly underneath IFT144; however, in the retrograde train, IFT144 is now at the same end of the repeating unit as IFTB1. Furthermore, not only are the two complexes completely flipped relative to each other, but IFTB is now wrapped around IFTA compared with the simple stacked relationship in the anterograde train. This results in a series of unanticipated interactions between IFTA and IFTB (Figure S2D). First, the two lobes of IFTB wrapping around IFTA2 means IFT139 (IFTA2) is now contacting IFT88 (IFTB1) on one side and IFT57/38 (IFTB2) on the other. Then, IFTA1 is contacted by two arms extending from the IFTB1 and IFTB2 subcomplexes. IFT81/74/52/46^inner^ (IFTB1) sits on top of the IFT144 tetratricopeptide repeat (TPR) domain, while the C terminus of IFT172^outer^ reaches out to contact the TPR domain of IFT140. This configuration means that IFT172^outer^ stretches between the two ends of the retrograde repeating unit.

Together, these results reveal that the retrograde train represents a fundamental rearrangement of the anterograde train that can only occur following complete disassembly at the tip. There is no mechanism in which the directional non-symmetric anterograde train can remodel into the non-directional 2-fold symmetric retrograde polymer without first disassembling. Underlining this, none of the lateral interactions in the anterograde train between adjacent IFTA or IFTB complexes are maintained into the retrograde train. Furthermore, the interactions between IFTA and IFTB in the retrograde train require extreme mobility relative to the anterograde conformation. Therefore, we conclude that retrograde trains are polymerized at the tip from individual IFT complexes.

### IFT144 forms a bridge that induces the formation of the retrograde train

These results show that the differences between the anterograde and retrograde trains are largely explained by how the IFTA and IFTB complexes interact with each other, rather than internal structural rearrangements of the complexes themselves. However, there are still some important conformational changes within both IFTA and IFTB between the two trains. We therefore wanted to understand how these conformational changes contribute to the remodeling process at the tip. We start by considering the IFTA complex since it is most important for establishing the architecture of the retrograde polymer.

**Figure 4:**
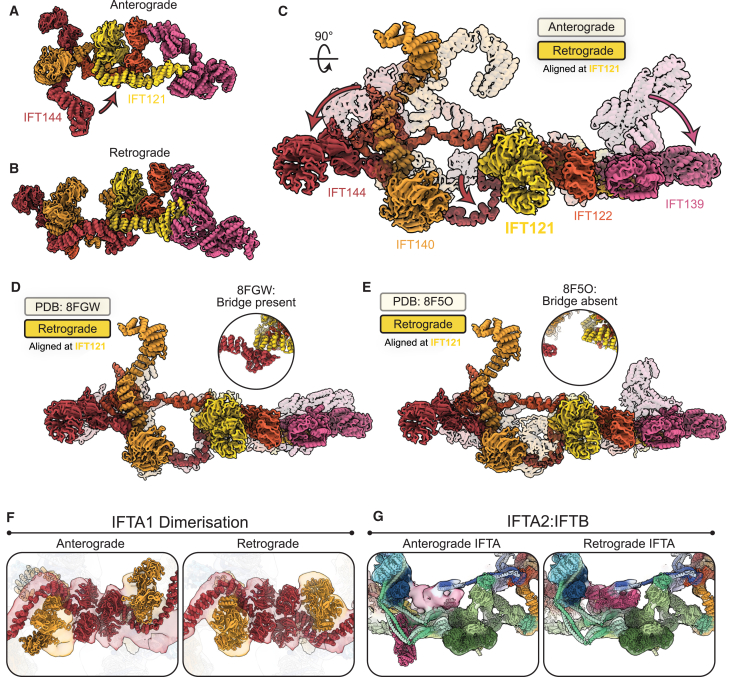
Internal stabilization of IFTA by the bridge is required for retrograde polymerization (A) A side view of a single IFTA complex from the anterograde train. Here, IFT144 (red) and IFT121 (yellow) are distant. (B) A single IFTA complex from the retrograde train, showing the formation of the bridge. The IFT144 (red) C terminus has now moved up to contact the IFT121 (yellow) TPR region, thus stabilizing the IFTA1 and IFTA2 lobes relative to each other. (C) A top view of a single IFTA complex from the retrograde train (opaque) superimposed with the anterograde train (transparent), showing the other changes in the two structures beyond the formation of the bridge. IFTA1 (IFT144/140, red/orange) swings around to be planar with IFTA2 (IFT121/122/139, yellow/dark orange/pink), and IFT139 (pink) straightens relative to the rest of the complex. (D) Comparison between the retrograde IFTA model (opaque) and PDB: 8FGW, a cryo-EM structure of purified human IFTA (transparent). Inset, the bridge is formed in this structure, with its formation resulting in IFTA1 and IFT139 adopting the same conformations as in the retrograde train. (E) Comparison between the retrograde IFTA model (opaque) and PDB: 8F5O, a cryo-EM structure of purified *Leishmania* IFTA (transparent). Inset, the bridge is not formed in this structure, resulting in a kinked IFT139 conformation and IFTA1 and IFTA2 not being in a retrograde conformation. (F) Left: the IFTA1 dimerization interface experimental density with the anterograde IFTA model docked in. The open conformation of IFTA1 prevents the dimerization of IFTA1, thus preventing polymerization. Right: the same view with the refined retrograde model docked in, showing the tight dimerization interface. (G) Left: close up of the interaction between IFT139 and IFTB. The experimental density is shown with the refined retrograde IFTB model docked in. The anterograde IFTA model shows IFT139 in the kinked conformation, resulting in large steric clashes that would prevent the formation of the IFTB2 polymeric interface. Right: the same view with the refined retrograde IFTA model docked in, showing how the clash is now resolved. See also Figure S2.

The recent IFTA single-particle structures showed that the IFTA1 and IFTA2 subcomplexes can possess a high degree of flexibility relative to each other when in solution.^24^^,^^25^ In the anterograde train, these two lobes are fixed into stable positions by the lateral contacts that build up the IFTA polymer.^13^ In the retrograde train, these stabilizing lateral interactions are lost, but the IFTA complexes still form rigid structures. We propose that this stabilization is generated internally in individual IFTA complexes through the formation of a bridge between IFTA1 and IFTA2 (Figures 4A and 4B).

IFTA1 and IFTA2 are normally linked to each other by the flexible IFT122 TPR domain. In the retrograde train, there is a second link between the two lobes, where the IFT144 (IFTA1) C terminus swings round to dock onto the side of IFT121 (IFTA2) (Figures 4A and 4B; Video S2). This bridge appears to stabilize IFTA1 and IFTA2 relative to each other. The movement in IFT144-TPR also shifts the IFT144 and IFT140 WD domains closer together (Figures 4C and S2E). Additionally, IFT139 moves from a bent to straight conformation relative to the rest of IFTA2 (Figure 4C). These conformational changes fix IFTA1 and IFTA2 in the planar conformation that allows them to run flat through the core of the retrograde train (Figures 2A and 2B).

Video S2. A morph between the IFTA complex in the anterograde and retrograde train models, showing the formation of the bridge between IFT144 (red) and IFT121 (yellow), related to Figure 4

To investigate this further, we compared our retrograde IFTA model to two single-particle IFTA structures that resolved IFTA1 and IFTA2 in a single map (as opposed to two independently masked maps). Significantly, in one of these structures IFT144 is docked to IFT121 to form the bridge (PDB: 8FGW),^26^ and in the other, IFT144 was undocked (PDB: 8F5O).^24^ The bridge-containing structure closely resembles IFTA in the retrograde train. The IFTA1 and IFTA2 lobes are planar, and the IFT144 and IFT140 WD domains are proximal (Figure 4D). In addition, IFT139 is in the straight conformation (Figure 4D), suggesting that the straightening of IFT139 could be allosterically linked to the formation of the bridge through the IFT121-TPR domain. Conversely, the structure without the bridge had the IFT144 and IFT140 WD domains more distant and IFT139 in the bent position (Figure 4E). In this structure, IFTA1 and IFTA2 are stabilized relative to each other through a unique interface between the IFT140 and IFT121 WD domains. Given that these structures are formed in solution, without the constraints imposed by other interactors in the retrograde train, these results suggest that the formation of the bridge is sufficient to stabilize the retrograde conformation of IFTA.

We next asked how the formation of the bridge affects the ability of IFTA to polymerize in the retrograde train. In the IFTA1 dimer interface, IFT144-WD domain dimerization is boxed in on each side by IFT140-WD. This multipartite interface is lost if IFT140-WD remains in the anterograde conformation (Figure 4F) or remains flexible. Conversely, the IFTA2 dimeric interface is mostly unaffected if the anterograde conformation is retained. However, if IFT139 remained in the anterograde conformation, it creates steric clashes with the IFTB2 complex sitting below it in the retrograde train (Figure 4G). We therefore conclude that internal stabilization of IFTA through the formation of the bridge locks the complex into the optimal conformation for polymerization. The formation of the bridge is therefore the key conformational change required for polymerization of the retrograde train.

### Flexible domains in IFTB become ordered to interact with IFTA

While the formation of the IFTA bridge appears to be important to initiate polymerization, IFTB is needed to complete the process given its presence at two of the three polymeric interfaces. Interestingly, two of the retrograde-specific interactions between IFTA and IFTB (Figure S2D) involve regions of IFTB that were flexibly tethered and thus unresolved in the anterograde train. Therefore, IFTA appears to stabilize retrograde conformations of IFTB.

First, in the anterograde train, the IFT172 C termini are flexible in the core of the train, with some involved in an interaction with IFT144/139 of IFTA.^13^ By contrast, the full length of both IFT172^inner^ and IF172^outer^ is resolved in the retrograde train. In IFT172^inner^, the C terminus forms an integral part IFTA2 dimeric interface, while in IFT172^outer^, it is docked to IFT140 from the same repeating unit (Figures 2D–2G). These interactions are both good candidates as the initial recruitment of IFTB^inner/outer^ to IFTA during remodeling since the IFT172 C termini extend out flexibly from the IFTB core.

The second flexible to ordered transition in IFTB involves a change in how IFT81/74 interacts with the rest of the IFTB complex. In the anterograde train, the N-terminal half of IFT81/74 sits on top of IFT88 and IFT70 in IFTB1,^13^^,^^32^ and the C terminus remained flexible and unresolved. In the retrograde train, IFT81/74 now sits at the end of the IFTB1 arm (Figures 2E, S2C, and S2D), via a biochemically characterized interaction between the C terminus of IFT81/74 and IFT52/46.^34^ This switch is accompanied by the IFT81/74^inner^ C terminus forming a long interface with IFT144 and IFT122 in IFTA1, down to the IFTB2 dimeric interface (Figure 1D). The IFT81/74 C terminus binding partners IFT27/25 and IFT22 are now resolved, interacting with IFTA and the IFTB2 polymeric interface, respectively.

Overall, the majority of the IFTB complex can be considered as a discrete unit during the IFT cycle. However, IFTA induces and stabilizes these two conformational changes when it rebinds IFTB during tip remodeling. We think this templates IFTB into the retrograde conformation and therefore generates the full complement of polymeric interfaces for retrograde polymerization.

### *In situ* XL-MS confirms retrograde train rearrangements

Since the new model of retrograde IFT trains diverges significantly from the anterograde train structure, we wanted to ensure that we had landed on the correct solution. To validate our model, we used *in situ* XL-MS. This technique involves chemically cross-linking whole cells and identifying peptides linked by the cross-linker.^35^^,^^36^ When using a cross-linker with a defined molecular length, such as disuccinimidyl sulfoxide (DSSO), this provides distance restraints that reveal whether two residues in a structure are modeled appropriately.

**Figure 5:**
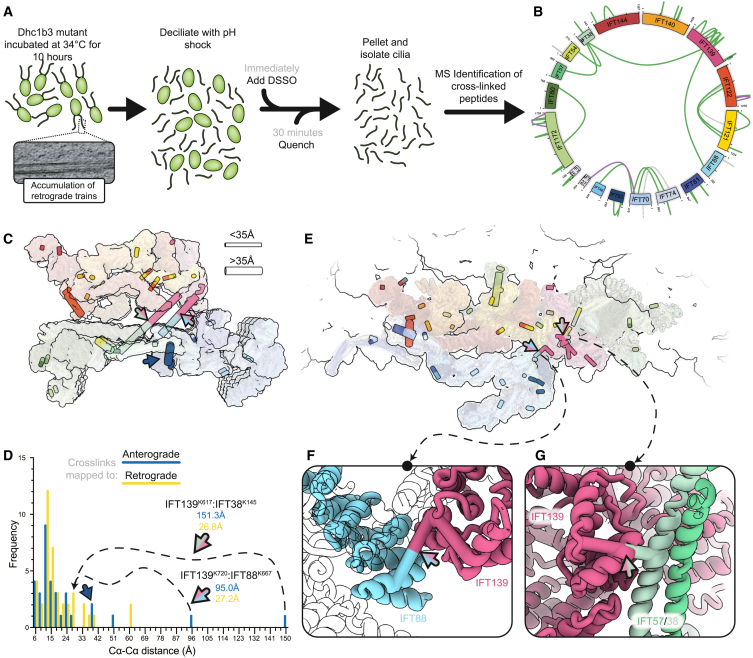
*In situ* cross-linking mass spectrometry validates the retrograde train model (A) Workflow used for *in situ* cross-linking mass spectrometry. Dhc1b-3 mutant cells were incubated at their restrictive temperature. Cells were deciliated with pH shock and DSSO cross-linked. The reaction was quenched, cilia were isolated, and the samples were processed for mass spectrometry analysis. (B) Circle diagram depicting the cross-links found within and between IFT complex proteins. Green lines indicate cross-links between residues <35 Å when mapped onto our retrograde model, purple lines are >35 Å, and gray dotted lines are between regions not modeled. (C) IFT cross-links were mapped onto the anterograde train model. Thin cross-links indicate distances <35 Å, thick cross-links >35 Å. Cross-links are color coded by protein. IFT139^K617^:IFT38^K145^ (pink/green) and IFT139^K720^:IFT88^K667^ (pink/blue) and IFT52 (dark blue) cross-links are indicated with arrows. (D) Histogram for the Ca-Ca distances when the IFT cross-links are mapped to the anterograde (blue) or retrograde (yellow) models. (E) IFT cross-links now mapped onto the retrograde train model. Thin cross-links indicate distances <35 Å, thick cross-links >35 Å. Cross-links are color coded by protein. IFT139^K617^:IFT38^K145^ (pink/green) and IFT139^K720^:IFT88^K667^ (pink/blue) cross-links are indicated with arrows. (F) Close-up view of the IFT139^K720^:IFT88^K667^ cross-link in the retrograde model. (G) Close-up view of the IFT139^K617^:IFT38^K145^ cross-link in the retrograde model. See also Figure S3, Figure S4, Figure S5, Figure S6.

**Figure S3:**
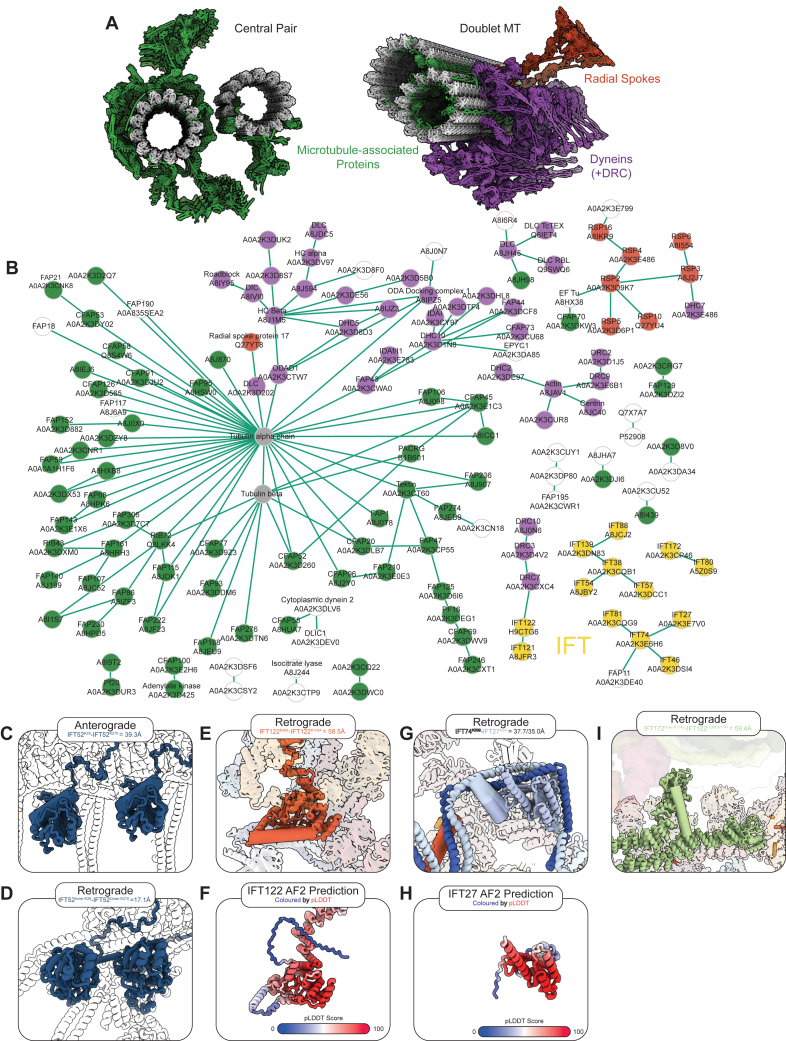
*In situ* cross-linking mass spectrometry of Dh1bc3 cilia, related to Figure 5 (A) Color coded PDB structures of the central pair (PDB: 7SQC and PDB: 7N61) or doublet (PDB: 8GLV) microtubules, showing tubulin in gray, microtubule-associated proteins in green, dyneins and associated complexes in purple, and radial spokes in red. (B) Full network of inter-protein cross-links identified in our *in situ* XL-MS experiment. Nodes are colored according to the classifications in (A). The majority of cross-links are for the proteins and complexes that made up the axonemal microtubule repeats. However, in yellow, a number of IFT protein cross-links are shown (IFT proteins with solely intra-protein cross-links are not shown here). Cross-links to non-IFT proteins could represent cargo interactions; IFT74 is interacting with FAP11, a TRP-like channel.^81^ White nodes correspond to proteins without a defined structural interaction to axonemal microtubules. Apparent protein pair level false discovery rate 4%. (C) Close up of the IFT52^K28^-IFT52^K270^ cross-link in the anterograde train, showing that it is best resolved between residues in the same copy of IFT52. The Ca distance is 39.3 Å. (D) The same cross-link in the retrograde model is now resolved with a Ca distance of 17.1 Å, as a result of remodeled IFTB lateral interactions bringing two adjacent copies of IFT52 closer together. (E) In the retrograde structure, the IFT122^K966-K1084^ Ca distance is 58.5 Å apart. (F) However, in the original Alphafold2 prediction, the loop of IFT122^K966^ is predicted to be flexible, thus explaining this cross-link in our structure. The original Alphafold2 prediction is shown here colored by the pLDDT score, with blue corresponding to lower confidence. (G) In the retrograde structure, the IFT74^K539^-IFT27^K2 or 4^ Ca distance is 37.7 and 35.0 Å, respectively. (H) The original Alphafold2 structural prediction of IFT22 colored by pLDDT score, showing that the N terminus of IFT27 is highly flexible. (I) The cross-link between IFT172^inner-K1342^:IFT172^outer-K1741^ is 59.4 Å in the retrograde structure. However, in the absence of stabilizing interaction at the IFTA2 dimeric interface, IFT172^inner^ would lose its anchor, thus explaining the cross-link.

Using the *dhc1b-3* cells to enrich for retrograde trains (Figure 5A), we identified 689 residue level interactions (488 self links, 201 hetero links) and 152 protein-protein interactions (Table S2). Since the most abundant protein in the cilium by an order of magnitude is tubulin, the majority of our cross-links correspond to tubulin or microtubule-associated proteins (Figures S3A–S3C). However, there were 48 cross-links for IFT complex proteins, including 9 protein-protein interactions (Figures 5B and S3B).

To determine if these cross-links validate our retrograde model, we first mapped them to our previous anterograde train structure (Figure 5C). The majority of IFT cross-links are between proteins in the same IFT subcomplex. Correspondingly, these cross-links are almost all shorter than 35 Å (Cα-Cα, generally taken to be the maximum permittable distance between two lysine residues linked by DSSO^37^^,^^38^) (Figure 5D). However, the two cross-links between different complexes (IFT139^K720^-IFT88^K667^ and IFT139^K617^-IFT38^K145^, between IFTA:B1 and IFTA:B2, respectively) are major outliers, with cross-link distances of 95 and 151 Å.

We next mapped the same cross-links to our retrograde model to see if these outliers are resolved (Figure 5E). These cross-links are consistent with the interactions made by IFTB wrapping around IFT139 in IFTA2 (Figure S2D). As such, these two residue pairs are both now under 30 Å from each other (Figures 5D, 5F, and 5G). Furthermore, a cross-link between IFT52^K28^ and IFT52^K270^ is 39 Å apart in the anterograde structure but is reduced to 17 Å in the retrograde structure due to the reduced lateral distance between IFTB1 subcomplexes (Figures S3C and S3D).

These results indicate that the rearrangements that modeled into our retrograde cryo-ET density do in fact reflect the native molecular conformation of retrograde IFT trains. Nevertheless, a small number of distance outliers still exist when we map the cross-links to the retrograde train (Figure 5D). However, these can all be explained by flexibility in the regions being cross-linked, as determined by Alphafold2 predicted local distance difference test (pLDDT) scores lower than 50 (Figures S3E–S3I).

To further confirm the veracity our of retrograde model, we performed integrative structural modeling with Assembline.^39^ This package integrates information from EM density, cross-links, and physical parameters (e.g., steric and connectivity restraints) to find optimal fits for user-supplied atomic models. It first performs systematic fitting of the input models as rigid bodies at different positions and orientations in the EM density. This already showed that our placement of various IFT subcomplexes and Alphafold2 predictions in our retrograde model corresponds to the statistically most favorable locations (see Methods S2).

**Figure S4:**
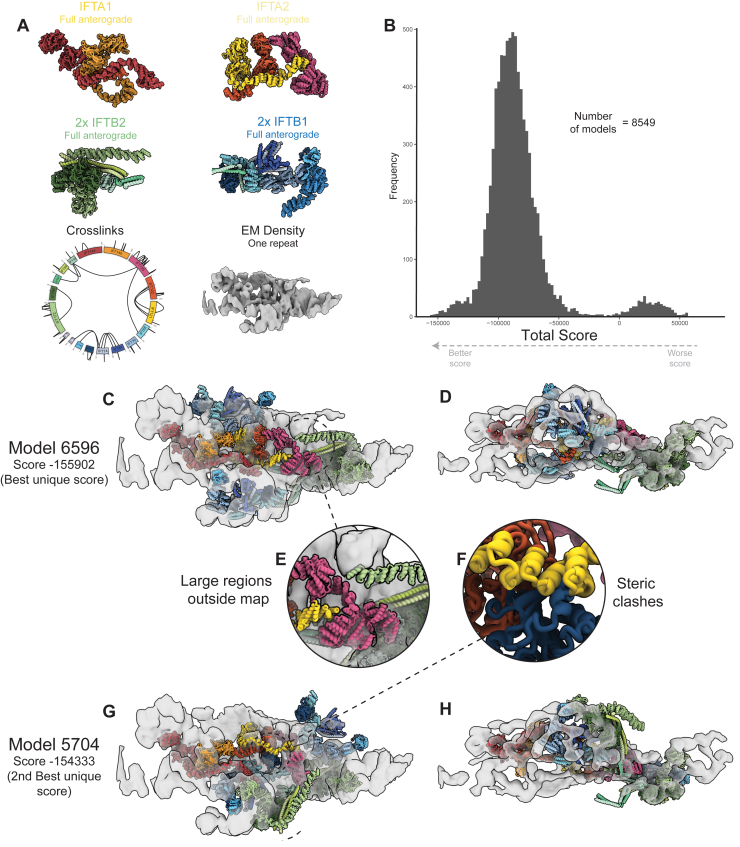
Assembline modeling of full anterograde complexes, related to Figure 5 (A) Input data for Assembline modeling of the full anterograde complexes. The rigid body models to fit corresponded to the full anterograde IFTA1, IFTA2, IFTB1, and IFTB2 complexes. Two copies of each IFTB subcomplex were used. These models were fit into one repeat of the composite retrograde EM density using the fit libraries shown in Methods S2 with the *in situ* XL-MS cross-links also used as restraints. (B) Histogram of the overall score Assembline calculates at the end of its global optimization run. Multiple modeling runs are started from random starting positions, with Monte-Carlo modeling run to optimize the experimental (e.g., EM fit and cross-links) and physical (e.g., clashes, connectivity) restraints. 8,549 independent runs were performed, with the overall score calculated based on the satisfaction of these restraints. Lower scores correspond to more optimal fits. The near Gaussian distribution of scores here is consistent with no good solutions being found since models have not converged at a high-scoring solution. (C and D) Orthogonal views of the best scoring model from the full anterograde IFT complexes run in Assembline. (E) The best scoring models from this setup have large domains outside the density. (F) The best scoring models from this setup have large steric clashes. Together with (B) and (E), this indicates that the Assembline global optimization is unable to find a good solution of these rigid bodies and the experimental restraints provided. (G and H) Orthogonal views of the second-best scoring model from the full anterograde IFT complexes run in Assembline.

**Figure S5:**
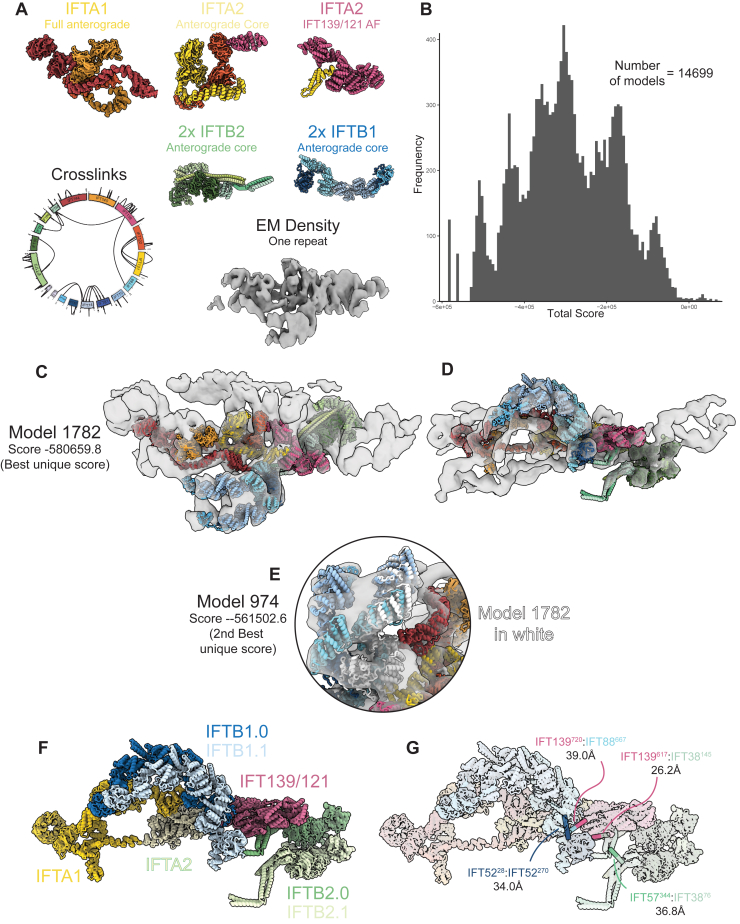
Assembline modeling of split anterograde complexes, related to Figure 5 (A) Input data for Assembline modeling of the split anterograde complexes. The input rigid models now have the potentially flexible regions removed, with IFTA2 split into two separate rigid bodies. The fit libraries for these updated rigid bodies to the same EM density as Figure S23 were used, and the *in situ* XL-MS restraints were also the same. (B) Histogram of the overall score Assembline calculates at the end of its global optimization run. Compared with Figure S23B, this histogram now shows convergence on better scoring solutions separated from the other worse scoring solutions. This indicates a good solution has been found. (C and D) Orthogonal views of the best scoring model cluster from this run. The model contains now steric clashes, with everything fitting inside the density. It corresponds exactly to our refined retrograde model, thus validating our model building. (E) The second-best scoring cluster in (B) matches the best scoring cluster identically, with the exception of the position of IFTB1^inner^ (IFTB1^inner^ from the best scoring cluster shown in white). The two positions of IFTB1^inner^ in these two clusters are themselves almost identical and correspond to the two peaks in Figure S22B. This alternative conformation lowers the score due to small steric clashes with IFT121 in IFTA2, which could easily be resolved with MDFF. (F) Demonstration of the seven rigid bodies used in the Assembline global optimization run. (G) Demonstration of the cross-links used in Assembline global optimization. All cross-links were given as inputs, but because the majority were between residues in the same rigid body, only these four were active. All four are satisfied or could be easily satisfied with flexible fitting now that the rigid bodies have been placed.

**Figure S6:**
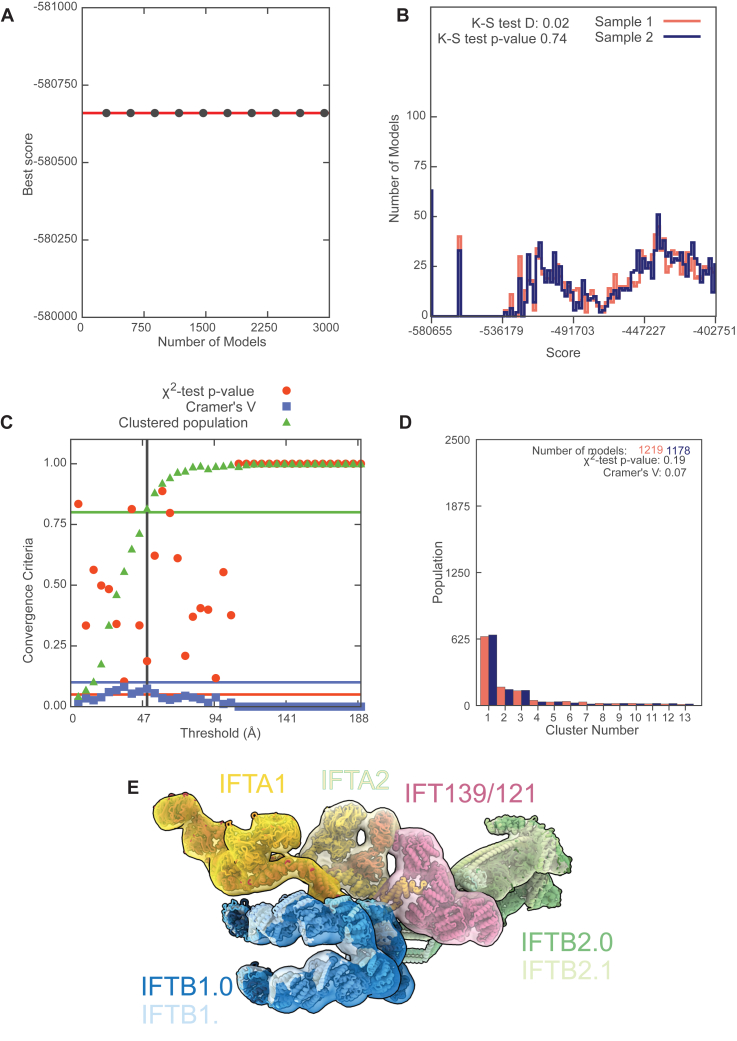
Sampling exhaustivity analysis of Assembline modeling, related to Figure 5 Results from sampling exhaustivity analysis^79^ performed on the top 20% of models from Figure S24B. (A) Convergence of model score. The best score does not improve as more models are added from a random subsample, indicating that a sufficient number of models have been generated. (B) Score distribution. The Kolmogorov-Smirnov two-sample *p* value is greater than 0.05, indicating that the distribution of scores when the models were randomly split into two groups is not significantly different. (C) Sampling precision analysis calculates a sampling precision of 50.0 Å. This is relatively low but is consistent with the Assembline methodology.^39^ (D) Distribution of models in clusters. (E) Probability localization densities of each rigid body in cluster 1 (D) closely represent the final Assembline (docked) and retrograde models. Cluster 1 precision is 36.983 Å.

Assembline then performs Monte-Carlo simulations from randomized starting states, thus ensuring that exhaustive sampling of all possible combinations of model poses is assessed. We started by considering each of the anterograde IFTA/B subcomplexes (IFTA1, IFTA2, IFTB1, and IFTB2) as rigid bodies. Assembline failed to generate any solutions without large steric clashes and entire domains sitting outside the density (Figure S4). This is consistent with our retrograde model, where there are conformational changes in each of the anterograde subcomplexes. Therefore, we repeated the process after splitting up the starting models into more rigid bodies. These simulations resulted in a cluster of high-scoring models that match exactly the localization of each of the subcomplexes in our retrograde model (Figure S5). Sampling exhaustiveness analysis confirmed that the conformational space has been sufficiently sampled (Figures S6A–S6D), and the resulting model represented a high-confidence solution (Figure S6E).

Taken together, these results confirm our analysis that the IFTA and IFTB complexes in the retrograde train undergo relatively minor internal rearrangements compared with the anterograde train but undergo large-scale remodeling relative to each other.

### Anterograde and retrograde trains present unique surfaces for cargo binding

We next asked whether our structures provide any insight into how anterograde and retrograde IFT trains perform their separate and opposing functions. The two trains bind to and transport unique sets of cargoes and motors. We reasoned that the conformational changes that occur during tip remodeling will result in changes in the surfaces available for cargo binding. This could mean the binding site of a cargo is available in one direction and inaccessible in the other. This would provide a mechanism to regulate unidirectional transport of specific cargoes within the bidirectional IFT system.

**Figure S7:**
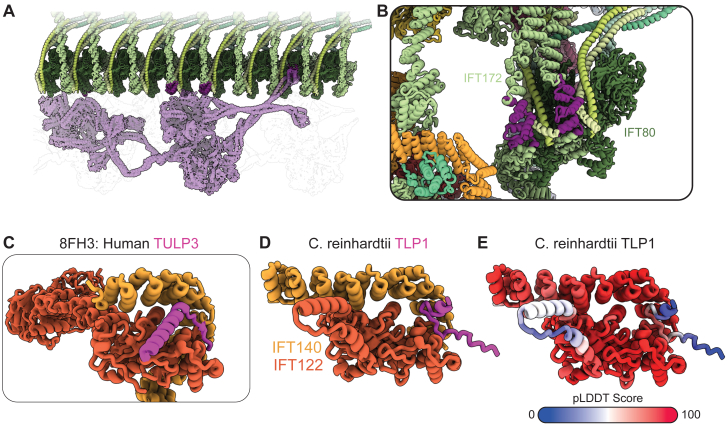
Cargo interactions in the retrograde train, related to Figure 6 (A) The binding site of autoinhibited dynein-2 on IFTB2 in the anterograde train. One dynein motor binds to multiple positions across multiple IFTB repeats. (B) The contact points of the dynein-2 motor domain in the anterograde train are now colored purple in the retrograde train. Remodeled lateral interactions mean that they are now much closer together, thus destroying the ruler-like anterograde binding sites. (C) The TULP3 (magenta) binding site in IFTA1 (IFT140/IFT122 orange/dark orange) from a single-particle structure of human IFTA, PDB: 8FH3. (D) Our Alphafold2 structure prediction of *C. reinhardtii* TLP1 and IFTA1 predicts the same interaction as the human homologs. (E) Same as (D), now colored by pLDDT score.

The best-characterized anterograde IFT cargo is cytoplasmic dynein-2. It is carried in an autoinhibited conformation through a composite binding site that stretches across six IFTB2 repeats^13^^,^^16^^,^^17^ (Figure S7A) before unloading at the tip. IFTB is only present in pairs in the retrograde train, meaning that the composite binding site is not recovered. Furthermore, some of the dynein binding sites are altered in the retrograde train, such as sites on adjacent IFT172 and IFT80 proteins with a ruler-like spacing in the anterograde train (Figure S7B). As such, autoinhibited dynein will be unloaded upon IFTB depolymerization and is unable to bind to retrograde trains. This will prevent aberrant removal of autoinhibited dynein from the tip as a cargo before it is activated.

**Figure 6:**
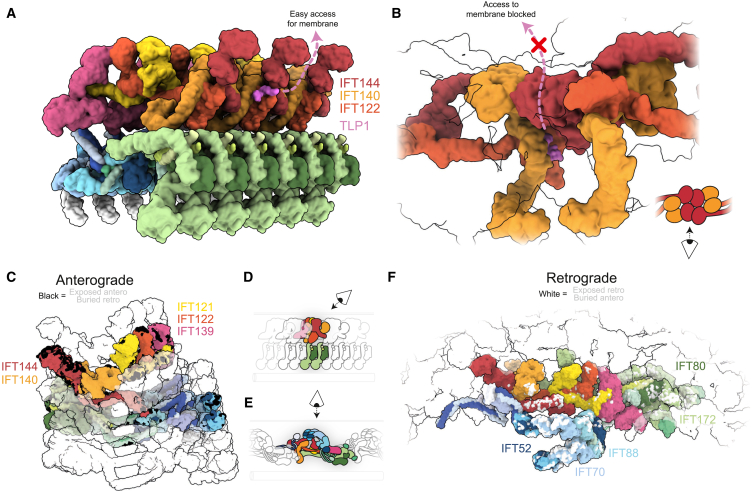
IFT train remodeling modifies cargo-binding sites (A) The TLP1 binding site allows easy access to the membrane in the anterograde train. (B) The same TLP1 interaction in the retrograde train is beneath the IFTA1 dimeric interface, making TLP1 inaccessible to the membrane. (C) One IFTA complex and two IFTB complexes are highlighted in the anterograde train. Black patches indicate regions that are surface-accessible in the anterograde structure but are buried in the retrograde train. (D) Cartoon representation to show the viewpoint of (C). (E) Cartoon representation to show the viewpoint of (F). (F) One repeating unit of the retrograde train is highlighted. White patches indicate regions that are surface-accessible in the retrograde structure but are buried in the anterograde train. See also Figure S7.

Another well-characterized cargo specific to anterograde trains is the TLP family. These proteins act as cofactors for membrane-cargo transport by IFT trains. Human TULP3 has been shown to bind to a pocket formed by IFT140 and IFT122 in IFTA,^25^^,^^26^ along with other members of the family. An Alphafold2 model of *C. reinhardtii* IFT140, IFT122, and the TULP3 homolog TLP1 predicts the same interface (Figures S7C–S7E). In the anterograde train, TLP1 is positioned with direct access to the membrane, allowing for interactions between the C-terminal domain and the membrane-based cargoes^25^^,^^26^ (Figure 6A). However, in the retrograde train, the formation of the IFTA1 dimer interface (Figure 2C) blocks off the route to the membrane, sequestering TLP1 on the microtubule-proximal side of the train (Figure 6B). Therefore, another mechanism for train-specific cargo interactions could be the removal of cargo-binding regions from their effector sites.

For the majority of IFT cargoes, however, the cargo-binding site remains unknown. Therefore, to identify potential train-specific binding sites, we identified all the regions of the anterograde train that become buried and inaccessible in the retrograde train, and vice versa. This revealed multiple patches on each train that are only accessible to cargoes in one direction. Significantly, all the membrane-proximal regions of the anterograde train are at least partially buried in the retrograde train (Figures 6C and 6D). Additionally, the formation of the bridge buries parts of IFT144 and IFT121 that are exposed and membrane-accessible in the anterograde train. Together, a large proportion membrane-proximal region of the anterograde train is set up to engage in train-specific cargo interactions.

In the retrograde train, the loss of anterograde polymeric interfaces and the conformational changes associated with the formation of the bridge have exposed a new surface on IFT140-WD and IFT144-TPR (Figures 6E and 6F). In IFTB2, the breakup of the anterograde polymer has exposed on one side the IFT172^inner^-WD domain and on the other side the IFT80^outer^-WD domain. There are also large patches on IFT88 and IFT70 of IFTB1 where the lateral interactions between adjacent repeats have splayed open. We therefore propose these represent potential cargo-binding interfaces that are regulated to produce bidirectional transport.

## Discussion

**Figure 7:**
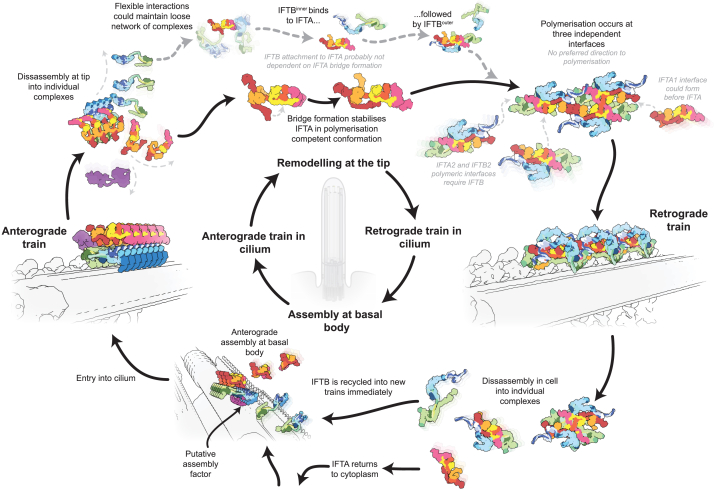
A summary of structural changes governing the IFT cycle Overview of our model showing the structural changes that occur during different stages of the IFT cycle.

Our results show that IFT complexes undergo a regulated cycle of conformational changes within the cilium to direct bidirectional transport. By comparing structural models of IFT complexes in anterograde and retrograde trains, as well as in solution, we now provide a broad understanding of this cycle (Figure 7; Video S3).

Video S3. An overview of our model of the structural changes that occur during the IFT cycle, related to Figure 7

### IFT complexes undergo a tightly regulated cycle of conformations in cilia

The cycle can be considered to start with polymerization of IFT complexes into anterograde trains at the basal body. Both IFTA and IFTB complexes are highly flexible in solution and are unable to polymerize *in vitro*, even at high concentrations.^24^^,^^40^ There must therefore be an exogenous factor at the basal body that promotes stabilization and polymerization of IFT complexes into the anterograde conformation. We previously proposed that an uncharacterized density in subtomogram averages of anterograde trains polymerizing at the basal body could be this packaging factor.^6^^,^^13^ Equally, IFT initiation is known to be dependent on post-translational modifications^41^ and regulated interactions with Rab-like GTPases.^42^^,^^43^ However, whether these effects act at the level of polymerization is not known.

By comparing the organization of the anterograde train and the retrograde train structures, we concluded that the anterograde train disassembles down into individual complexes once it has reached the tip. Nevertheless, we anticipate that some IFT complexes can stay loosely associated with each other through flexible interactions following anterograde train disassembly. There is one interaction between IFTA and IFTB retained from the anterograde train (IFT81/74 to IFT139), which is made flexible through the segmented coiled coil architecture of IFT81/74. We also identify roles for the flexibly tethered C terminus of IFT172^inner^ and IFT172^outer^ in linking IFTB to IFTA, consistent with previous results showing that IFT172 is required for turnaround at the tip.^44^ IFT complexes could also remain loosely associated at the tip through the disordered IFT144-IFT88 interaction,^25^^,^^45^ and even interactions with microtubules through IFT54-CH domain.^40^ These interactions would maintain a high local concentration of IFT proteins at the tip to aid retrograde polymerization. Furthermore, they could explain the observed association of IFT components from one anterograde train into only a small number of retrograde trains.^12^

It remains unclear what the trigger is for anterograde train depolymerization. One theory is that depolymerization is primed into the anterograde train structure.^9^^,^^12^^,^^13^^,^^14^ The anterograde train is characterized by interconnected lateral repeats, involving potentially compressed spring-like proteins. If a packaging factor loads them into this putative high-energy state at the basal body, it would take only a minor disturbance to induce relaxation and depolymerization. One candidate trigger is the detachment of the anterograde train from the microtubule,^9^^,^^46^ potentially induced by phosphorylation of kinesin-2 by *calcium-dependent protein kinase 1* (CrCDPK1)^41^ (*Chlamydomonas*) or ICK^47^ kinases.

Since adjacent repeating units in the retrograde train are internally stabilized, they do not rely on neighbors for their conformations. Instead, they stack together in simpler end-to-end interactions more amenable to self-assembly without exogenous factors. We propose that the internal stabilization of IFTA through the formation of the IFT144-IFT121 bridge initiates the retrograde polymerization. The formation of the bridge could be regulated by tip-specific post-translational modifications that mediate affinity for the regions of IFT144 and IFT121. IFT144 contacts IFT121 through a zinc-binding domain (ZBD), and it was previously reported that sequestration of zinc destabilized the complex.^26^ However, there is currently little evidence that zinc availability plays a physiological role in cilia. Nevertheless, a mutation to one of the cysteines in the ZBD leads to remodeling defects,^48^ consistent with its importance in the formation of the retrograde train.

We anticipate that IFTB mostly binds to IFTA prior to polymerization. Both IFTB^inner^ and IFTB^outer^ are involved in polymeric interfaces, suggesting that retrograde train polymerization is linked to the completion of the 2:1 IFTB:IFTA stoichiometry. Lateral interactions between IFTB^inner^ and IFTB^outer^ are like those found in the anterograde train, raising the question of what is stopping a third IFTB subunit from binding. We suspect that the lateral interactions between IFTB are relatively weak, and it is the interactions with IFTA that promote the dimerization of IFTB in the retrograde train.

At some point in the remodeling process, dynein-2 is activated and binds to the retrograde train. From our structure, we can identify regions on the sides and base of the retrograde train that could perform this role (Figures 2A, 2B, 2E, and 2F). It has often been speculated that IFTA directly binds to and activates dynein-2 at the tip, based on the absence of retrograde transport when IFTA is disrupted.^9^ However, our results suggest that these phenotypes are equally likely to be caused by the failure of retrograde train formation in the absence of IFTA. In fact, the sides and base of the retrograde train, where dynein would be expected to bind, are mostly made up of IFTB. Biochemical interactions between dynein-2 and IFT complex components have been identified, but they are all compatible with the binding mode found in anterograde trains.^49^^,^^50^^,^^51^ It is therefore unclear if they are still relevant in retrograde trains. We suspect it is more likely that dynein binds to a disordered region of the retrograde train since the 2-fold symmetry means that an ordered interaction orients half of the motors in the wrong direction. However, given recent results showing that dynein-2 is only fully activated when it interacts with IFT complexes,^52^ further work is required to understand dynein binding and activation by retrograde trains.

Once the retrograde train reaches the cell body, it disassembles into individual complexes again. Elegant photobleaching experiments in *Chlamydomonas* showed that IFTB components are retained at the basal body and recycled into new anterograde trains.^53^ However, IFTA complexes are not incorporated into new trains directly, instead returning to the cytoplasmic pool. Many IFTA complex proteins share homology to COPI coats and are thought to be involved in membrane trafficking.^54^ It was recently shown that purified IFTA complexes could fulfill this role by interacting with membranes using the “bottom” side of the complex (that is, the membrane distal side in the anterograde train).^24^ This side of the complex would then form the stacked interactions with the IFTB polymer in the anterograde train before engaging in a new set of interactions with individual IFTB complexes in the retrograde train. This highlights the structural diversity of the IFT complexes since this same surface is engaged in three unique sets of interactions at different stages of the IFT cycle. The IFT system is therefore intricately regulated to ensure each domain only engages in a specific set of interactions at each moment.

### Remodeling changes how retrograde trains interact with their environment

Based on our models, there are multiple structural mechanisms by which the IFT system could regulate its cargo interactions. First, train depolymerization destroys composite binding sites that span multiple repeats in the anterograde train, such as the one needed to bind dynein-2 as a cargo.^16^^,^^17^ Other large structural cargoes could be dependent on similar mechanisms, though the best-characterized of these (radial spokes, outer/inner dynein arms) are dependent on smaller cargo adaptors to bind to anterograde trains.^18^^,^^55^^,^^56^ The low stoichiometry of these large complexes relative to the repeating IFT complexes has so far prevented direct identification in cryoelectron tomograms.

Second, tip remodeling repositions IFT subcomplexes relative to their environment, thus regulating their accessibility for cargo binding. IFTA becomes more distant from the membrane, and IFTB flips so that the microtubule-proximal regions in the anterograde train are now facing the membrane. Exceptions to this include the C termini of IFT81/74 (along with IFT27/25/22) that have direct access to the membrane in both anterograde and retrograde trains. Multifunctional components of IFT trains may therefore be kept in position before and after remodeling as required.

Finally, internal rearrangements in IFT complexes means that some regions are buried and unavailable for interaction in one direction. Beyond the examples described, there could be subtler structural and allosteric changes that would only be visible with higher-resolution structures. Furthermore, all three of these effects will act in tandem with non-structural changes at the tip, including post-translational modification, local protein concentration, and small GTPase activities.

### Limitations of the study

The low resolution of our subtomogram averages limited us to domain-level interpretation of the retrograde train structure. We therefore are unable to visualize subtler, allosteric changes that could also play a role in remodeling. The influence of post-translation modifications of IFT trains is likely beyond even high-resolution subtomogram averaging and will require careful proteomic or cell-based studies. Furthermore, by primarily using a system in which the dynein motors are absent, we can only provide limited insights into the interaction with dynein and IFT trains.

## STAR★Methods

### Key resources table

**Table:** 

REAGENT or RESOURCE	SOURCE	IDENTIFIER
**Chemicals, peptides, and recombinant proteins**		

Gold Colloid solution 10nm	BBI solutions	#SKU EM.GC10/4
Tris base	Merck	93362
Hutner’s trace elements	Chlamydomonas Resource Center	https://www.chlamycollection.org/product/hutners-trace-elements/
Glacial Acetic Acid	Merck	A6283
K2HPO4	Merck	P3786
KH2PO4	Merck	P0662
NH4Cl	Merck	213330
MgSO4.7H2O	Merck	230391
CaCl2.2H2O	Merck	223506
Sucrose	Merck	S9378
MgSO_4_	Merck	M7506
Disuccinimidyl Sulfoxide	Cayman Chemical	9002863
N,N-Dimethylformamide	Thermo	326871000
Acetonitrile	Fisher chemical	10055454
Urea	Merck	U5378
Iodoacetamide	Merck	8047440025
DTT	Merck	43815
Trypsin/LysC protease mix MS-Grade	Pierce	A41007

**Deposited data**		

Structure of the IFT-A complex; IFT-A2 module	Hesketh et al.^25^	PDB: 8BBE
Structure of the IFT-A complex; IFT-A1 module	Hesketh et al.^25^	PDB: 8BBF
Human IFT-A complex structure	Jiang et al.^26^	PDB: 8FGW
Human IFT-A complex structure, with Tulp3	Jiang et al.^26^	PDB: 8FH3
Structure of Leishmania tarentolae IFT-A (state 1)	Meleppattu et al.^24^	PDB: 8F5O
IFTA complex in anterograde intraflagellar transport trains (Chlamydomonas reinhardtii)	Lacey et al.^13^	PDB: 8BDA
IFTB complex of anterograde Intraflagellar transport trains (Chlamydomonas reinhardtii)	Lacey et al.^13^	PDB: 8BD7
One repeating unit of the retrograde train	This study	PDB: 8RUY
Sub-tomogram average – Retrograde train composite consesus	This study	EMDB: 19515
Subtomogram average – masked refinement of IFTA1 in retrograde train	This study	EMDB: 19516
Subtomogram average – masked refinement of IFTA2 in retrograde train	This study	EMDB: 19517
Subtomogram average – masked refinement of IFTB1 (proximal region) in retrograde train	This study	EMDB: 19518
Subtomogram average – masked refinement of IFTB1 (distal region) in retrograde train	This study	EMDB: 19519
Subtomogram average – masked refinement of IFTB2 in retrograde train	This study	EMDB: 19520
Cross-linking mass spectrometry dataset	This study	ProteomeXchange PXD049195
Proteomics dataset	This study	ProteomeXchange PXD049260
Chlamydomonas reinhardtii genome assembly V6.1	Craig et al.^57^	Phytozome genome ID: 707

**Experimental models: Organisms/strains**		

Chlamydomonas: dhc1b3 mt+	Chlamydomonas resource center	CC-4422

**Software and algorithms**		

TOMOMAN V1.0	Khavnekar et al.^58^	https://github.com/wan-lab-vanderbilt/TOMOMAN
MotionCor2	Zheng et al.^59^	https://emcore.ucsf.edu/ucsf-software
cryoCARE v0.3	Buchjolz et al.^60^	https://pypi.org/project/cryoCARE/
Imod v4.11.7	Kremer et al.^61^	https://bio3d.colorado.edu/imod/
CTFFIND4 V4.1	Rohou and Grigorieff^62^	https://grigoriefflab.umassmed.edu/ctffind4
tom_deconv.m	Tegunov^63^	https://github.com/dtegunov/tom_deconv
Warp V1.1	Tegunov and Cramer^63^	http://www.warpem.com/warp/
M V1.1	Tegunov et al.^64^	http://www.warpem.com/warp/
Relion V3.1.3	Zivanov et al.^65^	https://relion.readthedocs.io/en/latest/
PEET V1.16	Nicastro et al.^66^	https://bio3d.colorado.edu/PEET/
LocSpiral	Kaur et al.^67^	https://github.com/1aviervargas/LocSpiral-LocBSharpen-LocBFactor-LocOccupancy
ChimeraX V1.6.1	Pettersen et al.^68^	https://www.cgl.ucsf.edu/chimerax/
Alphafold V2.3	Evans et al.^69^	https://github.com/google-deepmind/alphafold
Coot V0.9	Emsley and Cowtan^70^	https://www2.mrc-lmb.cam.ac.uk/personal/pemsley/coot/
NAMDinator	Kidmose et al.^71^	https://namdinator.au.dk/
ProteoWizard MSconvert V3.0.22314	Chambers et al.^72^	https://proteowizard.sourceforge.io/
xiSEARCH V1.6.745	Mendes et al.^73^	https://www.rappsilberlab.org/software/xisearch/
MaxQuant v2.4.2.0	Cox and Mann^74^	https://www.maxquant.org/
xiView	Graham et al.^75^	https://xiview.org
Assembline V1.0	Rantos et al.^39^	https://www.embl-hamburg.de/Assembline/

**Other**		

R3.5/1 Au200 Quantifoil grids	Quantifoil	R3.5/1 Au200
Hypersep C18 25mg Cartridges	Thermo	60108-376

### Resource availability

#### Lead contact

Further information and requests for resources and reagents should be directed to and will be fulfilled by the lead contact, Gaia Pigino (gaia.pigino@fht.org).

#### Materials availability

This study did not generate new reagents

#### Data and code availability

• Subtomogram average and atomic coordinate data have been deposited at the Electron Microscopy Databank and Protein Databank respectively, and are publicly available as of the date of publication. Accession numbers are listed in the key resources table.
• This paper does not report original code
• Any additional information required to reanalyze the data reported in this paper is available from the lead contact upon request

### Experimental model and study participant details

*dhc1b3* temperature-sensitive mutant *Chlamydomonas reinhardtii* cells (Chlamydomonas resource center, CC-4422, mt+) were grown in Tris-Acetate-Phosphate (TAP) media. Cells were maintained by bubbling in at 24°C with a 12 hour day/night cycle for 2-3 days before use. To induce the temperature sensitive mutation, a 400mL culture was moved to a shaking incubator set to 34°C for 10 hours and used directly for experiments.

### Method details

#### Cryo-EM grid preparation

4uL of temperature-treated *Dhc1b3* cells were added to Quantifoil R3.5/1 Au200 grids that had been plasma cleaned for 10s with an 80:20 oxygen:hydrogen mix (Solarus II Model 955, Gatan). Next, 1uL of 10nm colloidal gold solution (in phosphate-buffered saline; BBI solutions) was added, and incubated on the grid for 30 seconds. The grid was then back-blotted and plunge frozen in liquid ethane (Leica Automatic Plunger Freezer EM GP2).

#### Cryo-ET data acquisition

Tomographic tilt series images were collected on a Thermo Scientific Titan Krios G4 transmission electron microscope operating at 300kV. Movies were acquired in electron energy event representation (EER) format on a Thermo Scientific Falcon 4 direct electron detector fitted with a Thermo Scientific Selectrix X energy filter with a slit width of 10eV. A pixel size of 3.03Å/pixel, and a dose rate of 2.6e^-^Å^-2^s^-1^ and a defocus ranging from 2-4um was used for the 3s exposures at each tilt. The tilt series were collected with SerialEM,^76^ using a dose-symmetric acquisition scheme from 0° to ±30°, and then bidirectionally to ±60°. For full tilt series, this results in an accumulated dose of 104*e*^−^ Å^−2^.

#### Tomogram Reconstruction

Tomogram reconstruction was handled with the TOMOMAN pipeline.^58^ First, motion correction was performed with MotionCor2,^59^ with each EER movie split into 40 fractions. Averages from the odd and even frames were generated at this step if the resulting tomogram was to be used for cryoCARE denoising.^60^ The overall averages were dose weighted in Imod^61^ and run through CTFFIND4^62^ for CTF estimation. The tilt series were then fed through the imod/etomo pipeline for semi-automated gold fiducial alignment. Next, tomograms were reconstructed in etomo at bin4 for visualization and particle picking. For cryoCARE, training was performed on the odd and even frame averages. Each tomogram was trained individually, and then denoised. We found best results when the denoised tomogram was then fed through the tom_deconv filter.^63^

#### Particle picking

We identified retrograde IFT trains in our tomograms based on their position between the microtubule and membrane, and ∼45nm repeat. We manually picked the center point of each repeat (equivalent to the IFTA2 dimer interface in the final model) in a scattered model in the 3DMOD slicer.

#### Subtomogram averaging

For further details, we refer the reader to Methods S1. We initially obtained no signal in our refinements of the retrograde particles. Instead, we adopted two strategies to bootstrap the refinement. First, we boosted the signal to noise in our raw tomograms. To do this, we picked three microtubule doublets in each tomogram as a contour in 3DMOD, and extracted particles spaced every ∼10 tubulin dimers (80nm). We then extracted these coordinates as subtomograms in WARP V1.1^63^ with a box size of 80 pixels and a pixel size of 12.12Å (bin 4). We then performed 3D refinement in Relion V3.1,^65^ re-extracted the refined coordinates at bin 2, and re-refined in Relion. This typically resulted in reconstructions with a resolution around 17–20Å (this process was performed in batches corresponding to data collection sessions). We then performed local tilt alignments (Image Warp grid and volume warp grid) in M V1.1,^64^ which boosted the resolutions to around 12–13Å. This process optimizes the local tilt alignments throughout the tomogram, effectively using the microtubule doublets as a fiducial model. When we extract the retrograde particle coordinates, the subtomograms now have significantly improved signal to noise.

To further bootstrap the 3D refinement, we generated an average of all of the particles in each retrograde train without alignments (using PEET^66^). This provided enough signal to allow us to manually assign each train an estimate of the particle orientation. We recorded the angles needed to bring all the particles into the same orientation, then applied these angles to the star file when extracting the subtomograms in Warp.

We extracted the retrograde particles with a pixel size of 12.12Å bin4, and performed local 3D refinement in Relion (search angle/step both set to 3.7°). Because the particles were manually picked, we required a large translational search (8px/step 1px). As an initial reference, we used one of the intra-train averages used for angle estimations. Constraining the particle orientations around our manual estimation combined with the stronger signal in the M-refined subtomograms meant that Relion was now refining signal.

We next unbinned our particles to 6.06Å/px and performed a second local refinement in Relion. This time, we used a tighter mask for the central region (corresponding to IFTA2 in our final model). This meant that when we performed 3D classification without refinement using a longer mask we could remove particles with no features outside the central mask, indicating only noise had been refined in these particles. We then performed 3D refinement with the central mask again, this time applying C2 symmetry. This corresponds to the “Final IFTA2 map”.

To obtain maps for the remainding regions, we performed C2 particle symmetry expansion on the final IFTA2 map star file. We then performed masked alignments and, in the case of the IFTB maps, a further round of 3D classification. This resulted in a series of maps between 15Å and 28Å in resolution. We filtered each map with LocSpiral,^67^ then combined into a composite in ChimeraX.^68^

#### Model building

Once we had identified that the map contained two-fold symmetry, the features of IFTA1 and IFTA2 were quite obvious. The WD domains in the IFTA1 final map allowed us to fit in the IFT144 and IFT140 WD domains from our anterograde model, along with the IFT122 C-terminal domain they bind to. We added a full Alphafold2^69^ prediction of the IFT122 C-terminus compared to the truncated version from our anterograde model. For initial model docking we used Chimerax, and then performed manual modifications in Coot V0.9.^70^ For IFTA1, we split the WD domains of IFT144 and IFT140 from their TPR domains to fit each into the density independently, before joining back together. We then performed iterations of molecular dynamics flexible fitting (MDFF) in NAMDinator^71^ using default parameters. The largest manual modification we needed to make in IFTA2 was splitting IFT139 into two halfs (at a flexible hinge based on Alphafold2 pLDDT score) and docking the two halves back in.

For IFTB, we split our anterograde model into IFTB1 and IFTB2. We first located IFTB2 in our map through the characteristic crescent shape of the IFT172 domain. We identified this motif twice, and obtained a strong fit for the two IFTB2 (IFTB2^inner/outer^) domains in the initial rigid body fitting. We needed to perform manual modifications to the long IFT172 TPR domains to straighten them into the density. IFTB1 was located through the characteristic pitch of the two TPR superhelices IFT88 and IFT70. We removed IFT81/74 from the anterograde model since this did not fit the density. Instead, we performed an alphafold prediction of the IFT81/74 C-terminus in complex with IFT27/25 and IFT52/46. When aligned to IFT52/46 in the refined IFTB1^inner^ model, this fit IFT81/74/27/25 into the extra density at the end of the IFTB1 arm. We could then trace the remaining IFT81/74 helices down to the IFTB2 interface, including the region binding to IFT22. We do not identify the first four coiled coils or CH domain of IFT81/74. Manual modification was performed at hinges between coiled coils. In IFTB1^outer^, it appears that IFT81/74/27/25 fits into a similar density to that at the end of the IFTB^inner^ arm, however the path after that is not clear. There is an unfilled density in this region that likely corresponds to IFT81/74^outer^, but is at lower resolution and without clear features.

Model and density visualization was performed in ChimeraX.

#### Cross-linking mass spectrometry

A 1L culture of the *dhc1b-3 C. reinhardtii* cells was incubated at 34°C for 10 hours. The cells were pelleted at 800g for 10 minutes, and resuspended in 100mL 10mM HEPES pH7.5. The cells were then repelleted, and resuspended in 30mL HDMS buffer (10mM HEPES pH7.5, 1mM MgSO_4_, 1mM DTT and 4% sucrose). Deciliation was performed by adding 7.5mL 25mM dibucaine, immediately followed by 7.5mL HDMS supplemented with 5mM EGTA. This solution was immediately made up to 3.5% Dimethylformamide (DMF) to aid cross-link solubility. The solution was then made up to 1mM disuccinimidyl sulfoxide (DSSO), from a fresh stock in 20% DMF. The total time between addition of dibucaine and DSSO was ∼30 seconds. The solution was incubated at room temperature with agitation for 45 minutes. 100mM Tris pH7.5 was then added to quench the reaction, and the cell bodies were pelleted out through centrifugation at 800rcf for 10 minutes. Cilia were pelleted through a sucrose cushion of HDMS/25% sucrose, spinning at 12000g for 20 minutes. The pellet was resuspended in 1mL HDMS supplemented with 100mM NaCl. Four sample volumes of ice-cold mass-spectrometry grade acetone was added and the mixture was placed at -20°C for 10 hours. The mixture was then spun at 10000g for 10 minutes, and the pellet was air dried in a tissue culture hood.

#### Peptide preparation for mass spectrometry

The protein pellet was processed for mass spectrometry by in-solution digestion. Briefly, the pellet was resuspended in 8M urea at room temperature and cysteines were reduced with 2.5mM DTT and alkylated with 5mM iodoacetamide. Urea concentration was brought down to 2M by dilution in 50mM ammonium bicarbonate prior to addition of tryspin/LysC cocktail (1:50 weight/weight ratio, Pierce) for overnight digestion at room temperature. The peptides were desalted by with a C18 matrix (Hypersep C18 25mg, Thermo Scientific) and subsequently separated by size exclusion chromatography on a Superdex 30 3.2/300 increase column (Cytiva) equilibrated with 30% acetonitrile, 0.1% trifluoroacetic acid. 50μl fractions were collected and early eluting fractions were taken for crosslinking MS acquisitions by LC-MS.

The whole procedure was carried out twice, yielding samples for 2 datasets.

#### DSSO Crosslinking MS acquisition

Approximately 1ug of peptides were injected for each liquid chromatography-mass spectrometry (LC-MS) acquisition. The LC-MS platform consisted of a Vanquish Neo system (ThermoFisher Scientific) connected to an Orbitrap Eclipse Tribrid mass spectrometer (ThermoFisher Scientific) equipped with a FAIMSpro device operating under Tune 3.5.3886. Mobile phases consisted of 0.1% v/v formic acid in water (mobile phase A) and 0.1% formic acid in 80% acetonitrile/water v/v (mobile phase B). Samples were dissolved into 4% mobile phase B. The FAIMSpro device was set to standard resolution with a carrier gas flow of 4.6L/min. The samples were separated on an EASY-Spray PepMap Neo column (75 μm x 50 cm) (ThermoFisher Scientific). Peptides were separated on 110 minute gradients designed to match the hydrophobicity of the various SEC fractions, with linear separation ranges from 20%-40%B in 77 minutes (earliest fraction, most hydrophobic), down to 11%B to 35%B (latest fraction, least hydrophobic).

MS1 spectra were acquired with a resolution of 120,000 and automated gain control target set to 400% and 50ms maximum injection time. Source RF lens was set to 35%. Dynamic exclusion was set to single count in 60 seconds. The duty cycle was set to 2.5 seconds. A precursor charge filter was set to z=3-7. Precursors were selected based on a data-dependent decision tree strategy prioritizing charge states 4–7 and subjected to stepped HCD fragmentation with normalized collision energies of 18,24,30.^77^ MS2 scans were acquired with a normalized gain control target of 750% with maxmum injection time of 150ms and an orbitrap resolution of 60,000. For each fraction, multiple injections were carried out with multiple FAIMS control voltages, with the first 3 injections being performed at -45V, -55V and -65V separately. For dataset 2, automatic gain control in MS1 was set to 250%, dynamic exclusion to single count in 30 seconds. Additional injections with a dynamic exclusion of single count in 10 seconds were carried out in dataset 2 whenever possible.

For database construction for dataset 1, 500ng of the unfractionated sample was injected and acquired with a proteomics method. The LC gradient was in this case 2-35% B over 120 minutes. MS1 was carried out in the orbitrap with a resolution of 120,000, automaic gain control target of 100% and 25ms maximum injection time. The RF lens was set to 30%. The charge state filter was set to 2-5 and dynamic exclusion to single count in 45 seconds. MS2 was carried out in the linear ion trap in rapid mode with HCD fragmentation at normalized collision energy of 30. The duty cycle was 2 seconds and no FAIMS device was employed. For dataset 2, each fraction was injected instead, and proteomics was carried out with the same method, but with the FAIMS device set to multiple CV of -45/-65 each with a duty cycle of 1.25 seconds.

#### DSSO Crosslinking MS data analysis

Raw files were converted to mgf format using ProteoWizard MSconvert (version 3.0.22314).^72^ A recalibration of the MS1 and MS2 m/z was conducted based on high-confidence (<1% false discovery rate) linear peptide identifications using xiSEARCH (version 1.6.745). Crosslinking MS database search was performed in xiSEARCH (version 1.7.6.7).^73^ The database was constructed using the top 600 proteins by iBAQ from a proteomic data analysis of bulk, unfractionated peptides (dataset 1) or all fractions (dataset 2). Proteomics analysis was carried out using MaxQuant v2.4.2.0^74^ and the reference *C. reinhardtii* from UniProt downloaded on 24/11/2023 (corresponding to the phytozome *C. reinhardtii* genome assembly version 6.1^57^). Precursor mass error tolerance was set to 3ppm and MS2 error tolerance to 5ppm. The search included methionine oxidation, asparagine deamidation as variable modifications. The DSSO crosslinker was defined as cleavable, and DSSO-NH2, DSSO-OH modifications were included in the search as variable modifications searched on linear peptides only.^78^ The search was set to account for noncovalent gas-phase associations. Prior to FDR estimation, search results were filtered to only include peptide spectra matches with at least 2 crosslinker-containing fragments on both peptides. Results were filtered to 3% FDR at the residue pair level using xiFDR (version 2.1.5.2) and the “boost” feature to optimize thresholds at the lower error levels was enabled to maximise heteromeric crosslinks. Results were exported in mzIdentML format and uploaded to xiview.org ^75^ for visualization.

#### Assembline modelling

For further details, we refer the reader to Methods S2. Assembline v1.0 was installed as a conda environment.^39^ Input models were prepared from the anterograde IFTA (PDB: 8BDA) and IFTB (PDB: 8BD7) structures.^13^ We cropped our composite retrograde map down to a single repeating unit to minimise computational requirements, and converted our cross-links to XlinkAnalyzer format. We used identical settings for the full anterograde complex and core anterograde complex runs. First, fit libraries were generated using the Assembline efitter pipeline. 80,000 positions were calculated, with a clusterAngle 4, clusterShift 3, radius 200, and inside threshold 0.3. The results from these runs are shown in Figures S20–S22. For global optimisation, we specified 2 copies each of IFTB1 and IFTB2, and one copy each of IFTA1 and IFTA2. The rigid bodies corresponded to the subcomplexes used in the fit library generation. Simulated annealing was performed with the following regime: Temp 75000/1000 steps; 30000/800; 10000/800; 5000/500; 1000/500; 300/300. The following restrain weights were used: Connectivity 1, X-link 30, discrete 10000, excluded volume (steric) 10, EM 1000. Following global optimisation, sampling exhaustivity analysis was performed on the top 20% scoring models with the Assembline wrapper for the imp-sampcon toolkit.^79^

### Quantification and statistical analysis

Resolution estimates of subtomogram averages are based on the 0.143 gold standard FSC criterion,^80^ with FSC plots found in Figure S1. For integrative structural modelling in Assembline, sampling exhaustivity analysis was performed on the top 20% scoring models using the imp-sampon toolkit,^79^ with the results shown in Figure S6

## Supplemental information

Methods S1. Description of steps for subtomogram averaging and AlphaFold modeling of the retrograde train, related to Figure 1

Methods S2. Assembline modeling fit library scores, related to Figure 5

Table S1. Cryo-EM data collection parameters and model statistics, related to Figure 1

Table S2. A list of all cross-links identified in our Dhc1b3 datasets after FDR filtering, related to Figure 5
