# Supplementary material for: Extensive structural rearrangement of intraflagellar transport trains underpins bidirectional cargo transport
Source: Cell. 2024 Aug 22;187(17):4621–4636.e18. doi: 10.1016/j.cell.2024.06.041 (PMC11349379; doi:10.1016/j.cell.2024.06.041)
Supplement: Methods S1. Description of steps for subtomogram averaging and AlphaFold modeling of the retrograde train, related to Figure 1 [file mmc1.pdf]

Cell, Volume *187*

Supplemental information

Extensive structural rearrangement of intraflagellar transport trains underpins bidirectional cargo transport

Samuel E. Lacey, Andrea Graziadei, and Gaia Pigino

Methods S1

Description of steps for Subtomogram averaging and Alphafold modelling of the retrograde train, related to Figure 1.

Using microtubule doublets as a fiducial for local alignment of tomograms

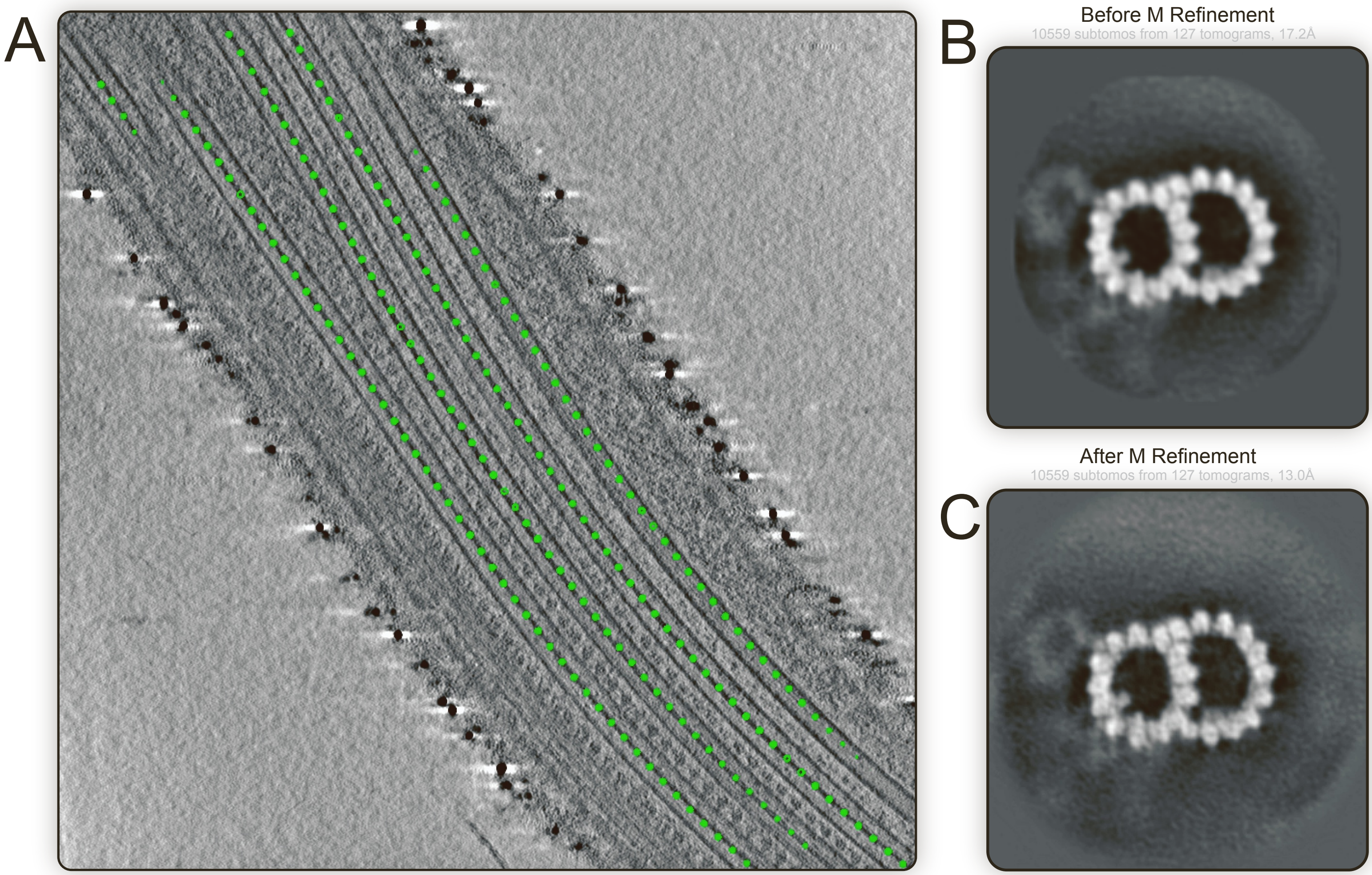

A – Doublet microtubules were picked in each of the tomograms prior to retrograde train STA. Green dots represent refined particle coordinates after subtomogram averaging. MT doublet averages were used for local refinement of tilt angles, so were sampled from the across the entire tomogram.
B – Doublet microtubules were subjected to STA in batches, yielding reconstructions of ~15-17Å
C – Doublet microtubule STA averages were used for M refinement, improving the resolution substantially. Averaging of retrograde trains was only possible after the local alignment of tilt angles, thus increasing the signal to noise in subtomograms.

Subtomogram averaging processing workflow

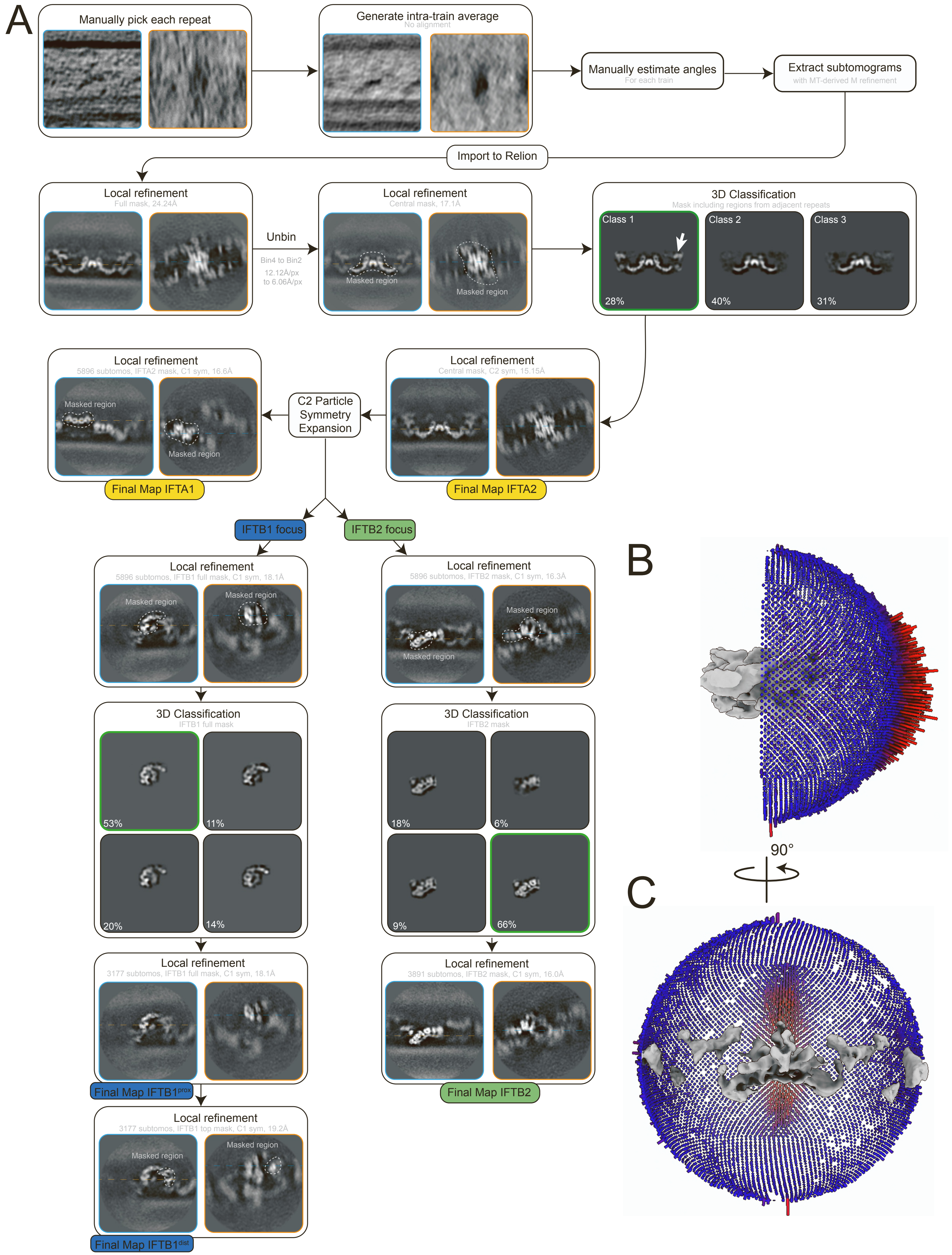

A – Particles from each train were picked manually. Intra-train averages without alignments were then generated and used to manually estimate angles to bootstrap local refinements. In Relion, local refinement was first performed on the entire structure at bin4. A bin2 alignment was then performed with a tighter central mask, and 3D classification was performed with an original wider mask. This separated good from bad particles by removing particles that had no signal outside of the refinement mask in the previous step. Further local refinements were performed on the different regions of the map to generate the components of the final composite map.
B – Representation of angular distribution of subtomograms contributing to the final IFTA2 map from an end-on view (as if looking down the microtubule). More particles are indicated by increasingly red bars. The majority of our subtomograms were side views of the filament, resulting in anisotropic resolution.
C – Side view of C, as if looking along the microtubule.

Starting structures for model building

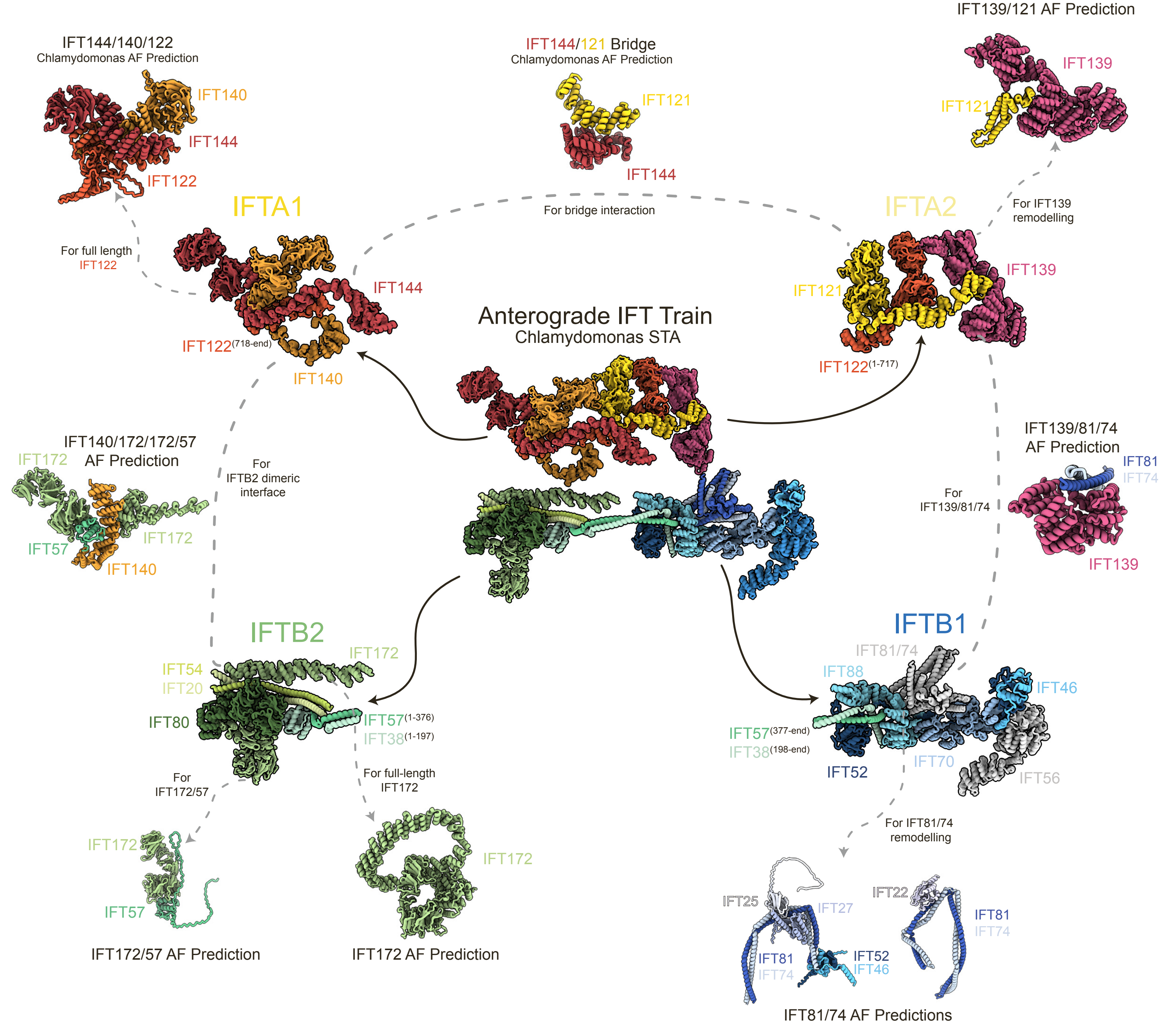

An overview of all the input structures we used to model the retrograde IFT train. We started with the structure of the entire anterograde IFT train (center), and split it into its four constituent subcomplexes (IFTA1, IFTA2, IFTB1, IFTB2). These models were supplemented with Alphafold2 multimer predictions ("AF prediction"); pLDDT and PAE plots for each prediction are provided in subsequent figures.

Alphafold predictions of IFTA used for modelling

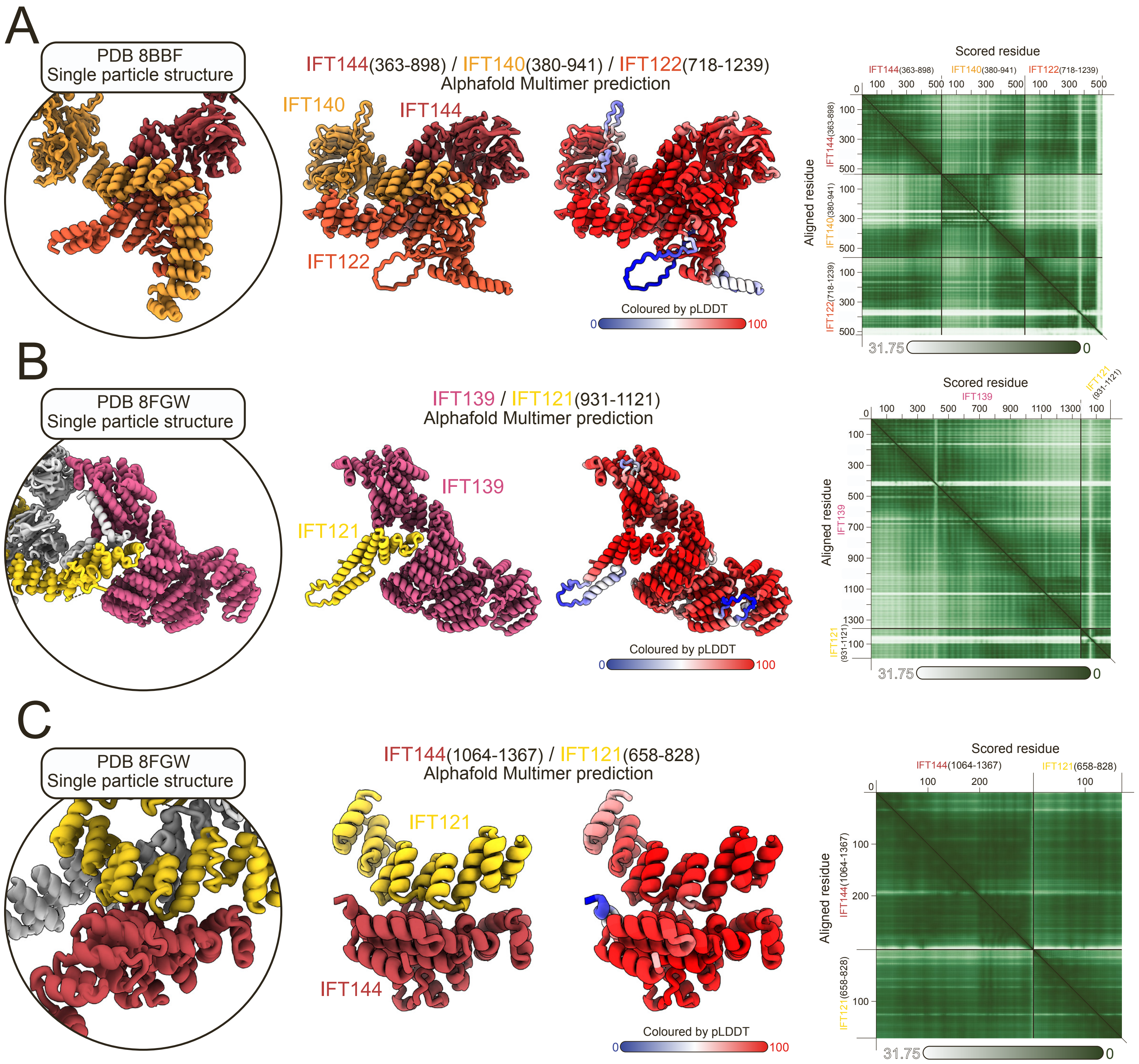

A – Structures of IFTA1 used to model the retrograde train. Left, Single particle structure of Human IFTA1 (PDB 8BBF, IFT144 (red), 140 (orange) and the C-terminus of IFT122(red-orange)). Center left, Alphafold2 prediction of C. reinhardtii IFT144$^{363\text{-}898}$/IFT140$^{380\text{-}941}$/IFT122$^{718\text{-}1239}$ coloured by subunit, showing high structural similarity to the single particle structure. This model was used to fill in the C-terminus of IFT122, in complex with IFT144/140, since it was left partially unmodelled in our anterograde structure. Center right, prediction coloured by pLDDT (predicted local different test) score. Higher confidence residues are coloured red, lower confidence white to blue. Right, pairwise pAE (predicted aligned error) plot, indicating the confidence of the location of each residue in the structure compared to each other residue. Lower pAE score (green) is higher confidence.

B – Structures of IFT139 (pink) in complex with IFT121 (yellow) in IFTA2. Left, single particle structure of Human IFTA (PDB 8FGW), focussed on the IFTA2 region. (IFT139, pink, IFT121, yellow, remaining subunits, grey). Center left, Alphafold2 prediction of C. reinhardtii IFT139/IFT121$^{931\text{-}1121}$, showing high structural similarity to the human single particle structure. This was used to model IFT139, so that we did not need to cut the protein into two rigid bodies manually. Center right, prediction coloured by pLDDT (red is higher confidence). Right, pairwise pAE plot (green is higher confidence).
C – Structures of the bridge interaction between IFT144 (red) and IFT121 (yellow). Left, single particle structure of Human IFTA (PDB 8FGW25), focussed on the bridge region. Center left, Alphafold2 prediction of C. reinhardtii IFT144$^{1064\text{-}1367}$/IFT121$^{658\text{-}828}$, showing high structural similarity to the single particle structure. This was used to model the bridge interaction. Center right, prediction coloured by pLDDT (red is higher confidence). Right, pairwise pAE plot (green is higher confidence).

Alphafold predictions of IFTB2 used for modelling

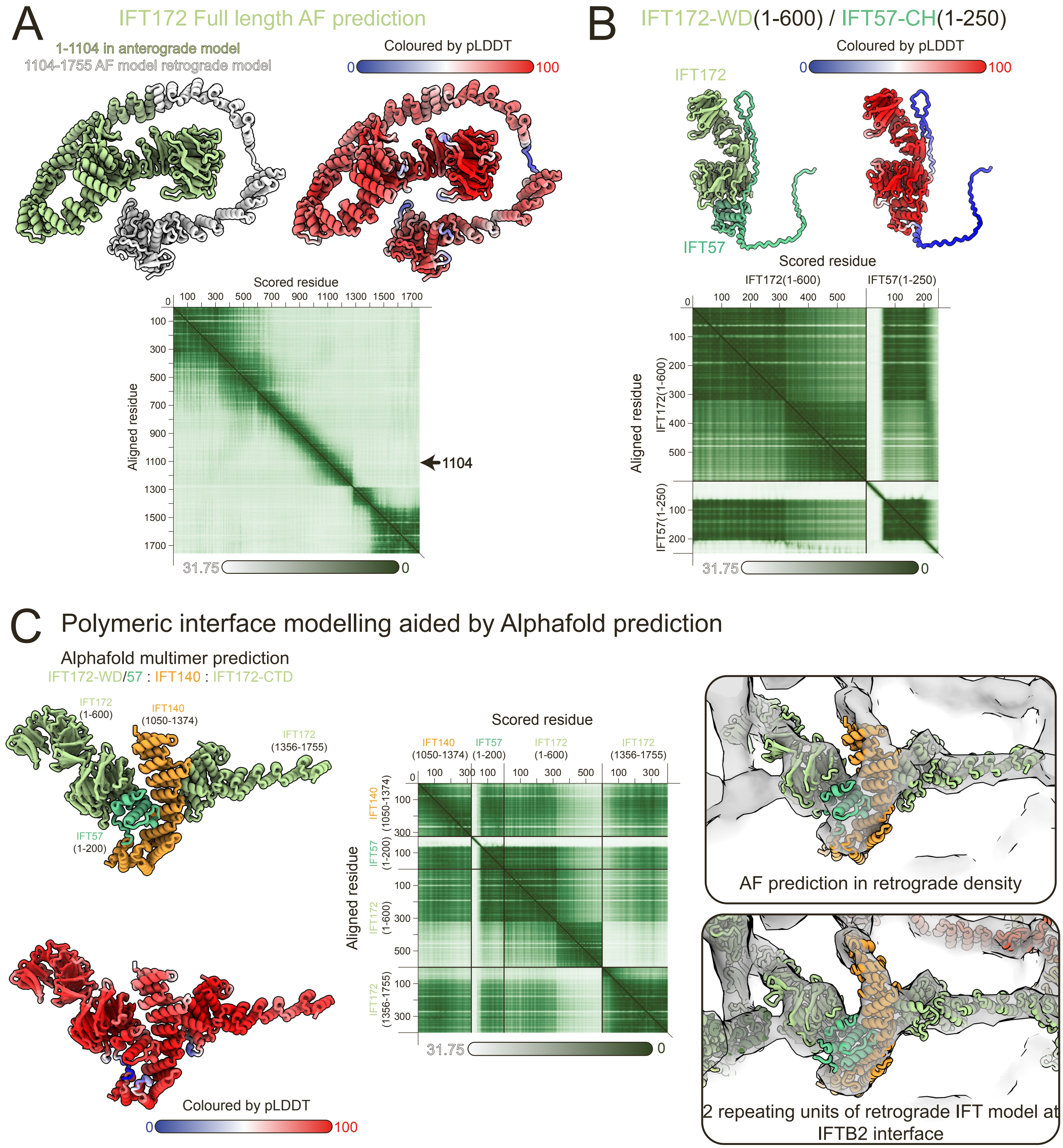

A – Alphafold2 prediction of IFT172 full-length. This was used to model the C-terminus of IFT172 that was not present in the anterograde model. Top left, prediction coloured by region present (green) or absent (white) in the anterograde model. Top right, prediction coloured by pLDDT (red is higher confidence). Bottom, pairwise pAE plot (green is higher confidence).

B – Alphafold2 prediction of IFT172$^{1\text{-}600}$/IFT57$^{1\text{-}250}$. These regions were selected based on the previously identified interaction between IFT172-WD and IFT57-CH. This prediction was used to model the WD-domain of IFT172 and its interaction with IFT57-CH domain. Top left, prediction coloured by subunit. Top right, prediction coloured by pLDDT (red is higher confidence). Bottom, pairwise pAE plot (green is higher confidence).
C – Novel Alphafold2 prediction of IFT172$^{1\text{-}600}$/IFT57$^{1\text{-}200}$/IFT140$^{1050\text{-}1374}$/IFT172$^{1356\text{-}1755}$. This was used to build and validate the IFTB2 polymeric interface. Top left, prediction coloured by subunit. Bottom left, prediction coloured by pLDDT (red is higher confidence). Center, pairwise pAE plot (green is higher confidence). Top right, the prediction docked into the retrograde density for the IFTB2 polymeric interface. Bottom right, the same view as above, showing two repeating units of the final retrograde model.

Alphafold predictions of IFTB1 used for modelling

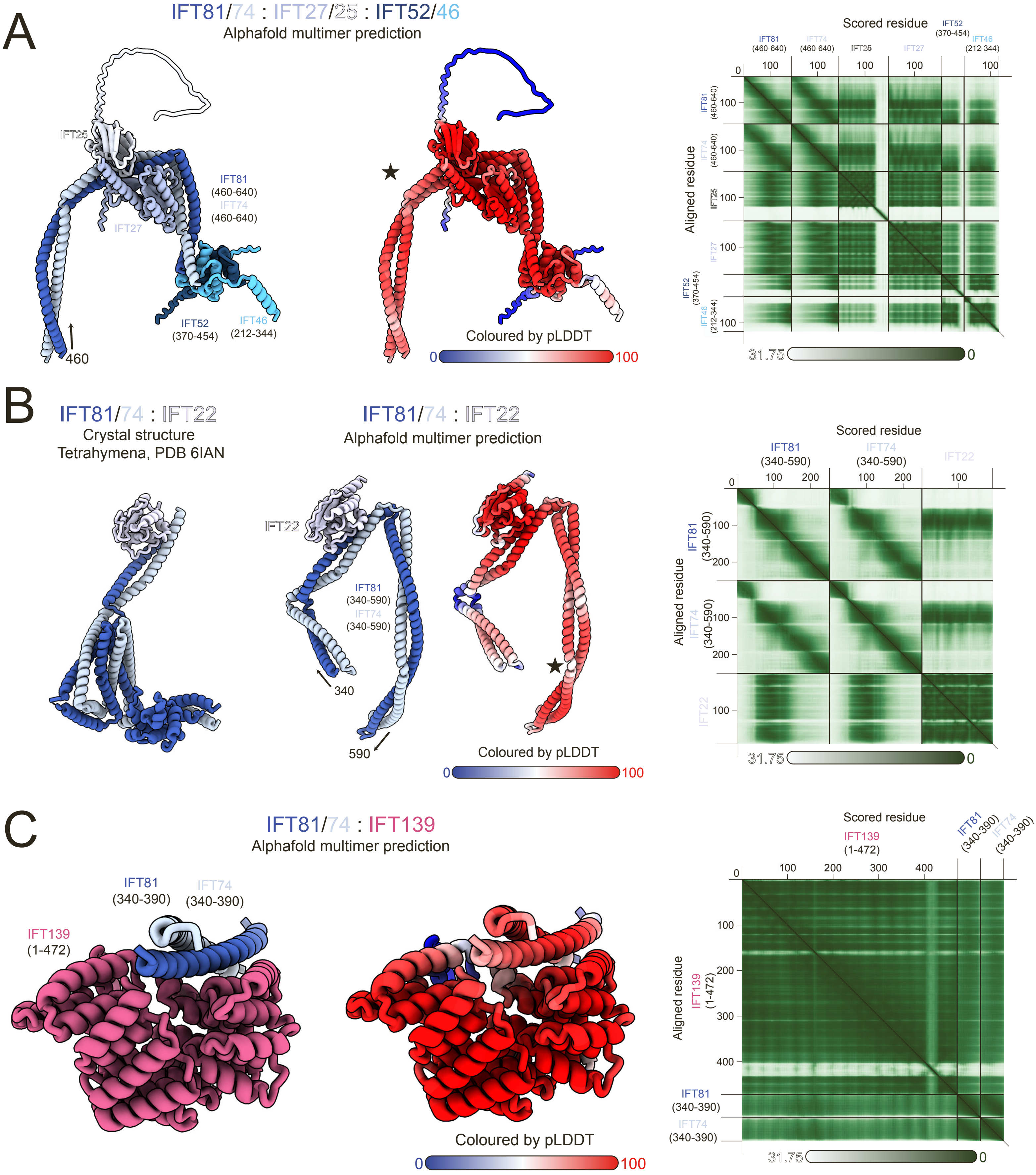

A – Alphafold2 prediction of IFT81$^{460\text{-}640}$/IFT74$^{460\text{-}640}$/IFT27/IFT25/IFT52$^{370\text{-}454}$/IFT46$^{212\text{-}344}$. These regions were selected based on previously identified interactions. This was modelled into the density at the end of IFTB1inner, corresponding to the biochemically identified interaction first established by Taschner et al. Left, prediction coloured by subunit. Center, prediction coloured by pLDDT (red is higher confidence).

Right, pairwise pAE plot (green is higher confidence). Star indicates flexible hinge also seen in overlapping region in B

B - Alphafold2 prediction of IFT81$^{340-590}$/IFT74340-590/IFT22. This was use to model the IFT81/74/22 complex at the IFTB2 polymeric interface. Left, Crystal structure of equivalent region from Tetrahymena. Center, Alphafold2 prediction coloured by subunit and by pLDDT (red is higher confidence). The crystal structure and Alphafold2 model are identical in this region, and the Alphafold2 model was used since it uses the Chlamydomonas sequence. Right, pairwise pAE plot (green is higher confidence).

C – Alphafold2 prediction of IFT81340-390/IFT74340-390/IFT1391-472. This was used to model the most N-terminal segment of IFT81/74 identified in our density. Left, prediction coloured by subunit. Center, prediction coloured by pLDDT (red is higher confidence). Right, pairwise pAE plot (green is higher confidence).

Step 1: WD domains resembling IFTA1 identified

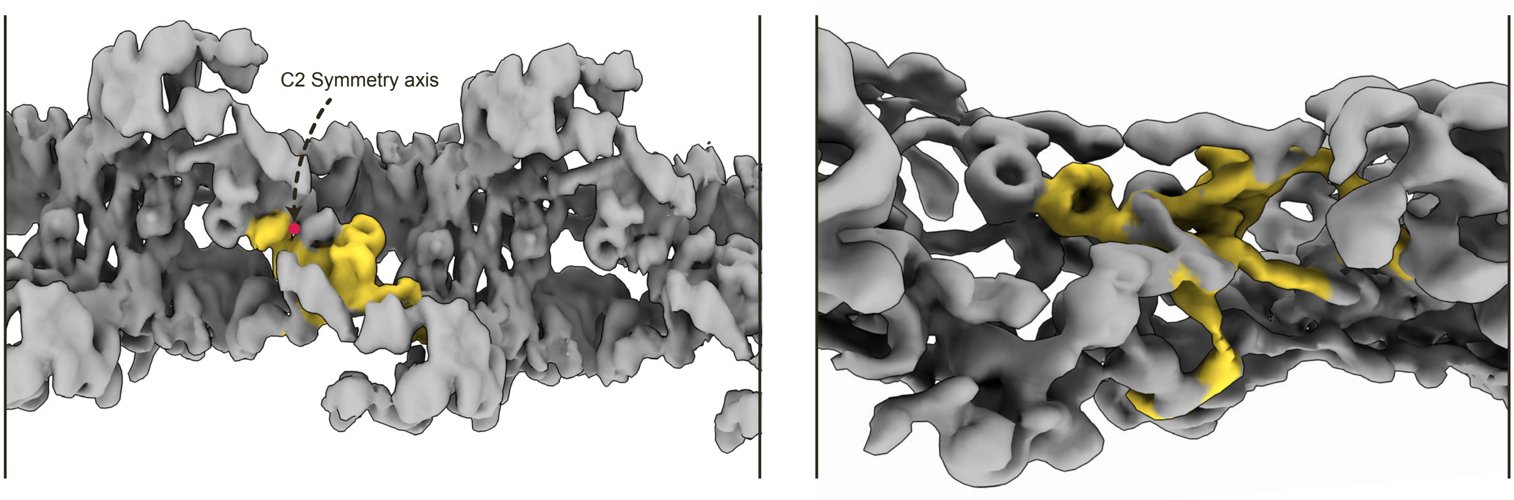

We first identified tandem WD domains at a C2 symmetry axis (yellow region). The presence of this symmetry pre-determined the stoichiometry of the model in this region.

Step 2: Fit and adapt existing IFTA1 models into density

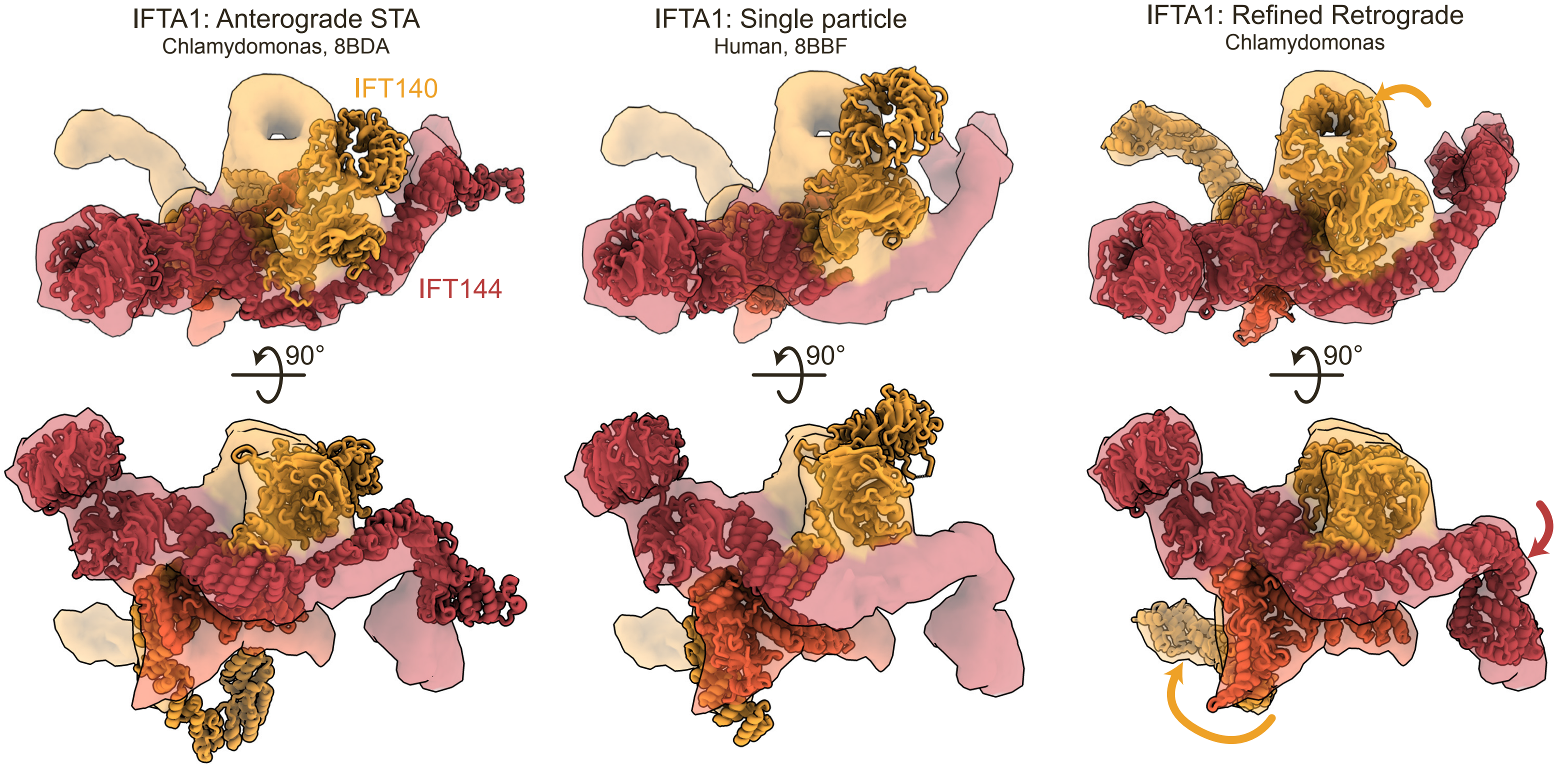

The only region of the IFT complex to contain tandem WD domains is IFTA1. We fit IFTA1 from our anterograde structure (left) and from single particle structures (center, 8BBF shown). They showed good overall agreement with the density, and required only minor structural modifications to fit into the density in our final retrograde model (right).

Step 3: Expand model into repeating density

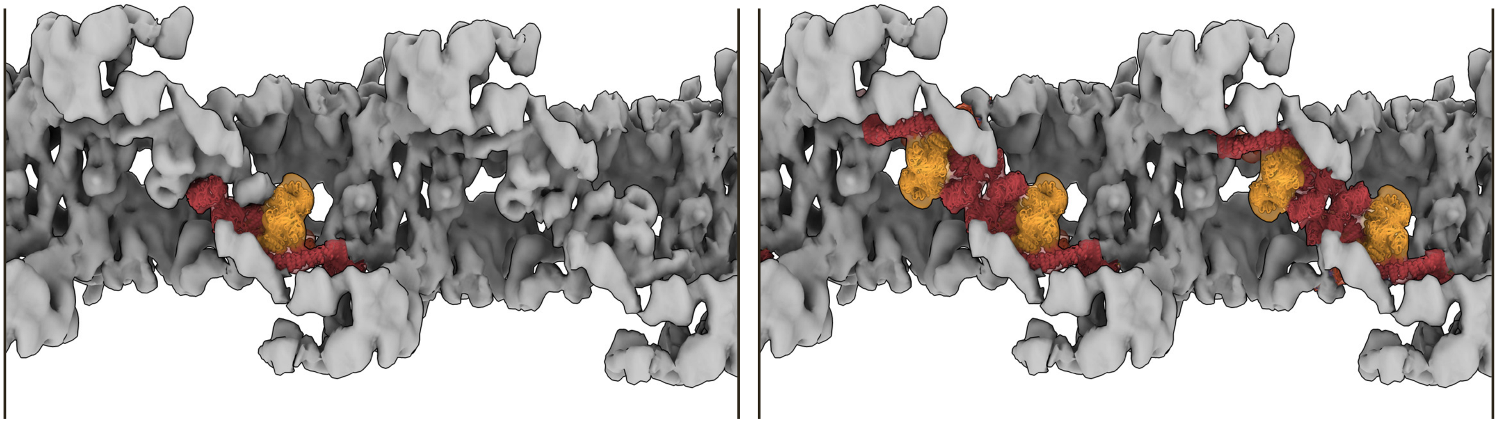

We expanded this model into the adjacent repeats, thus narrowing the possible regions that other domains could fill.

Step 4: Additional WD domain identified as IFT121 in IFTA2

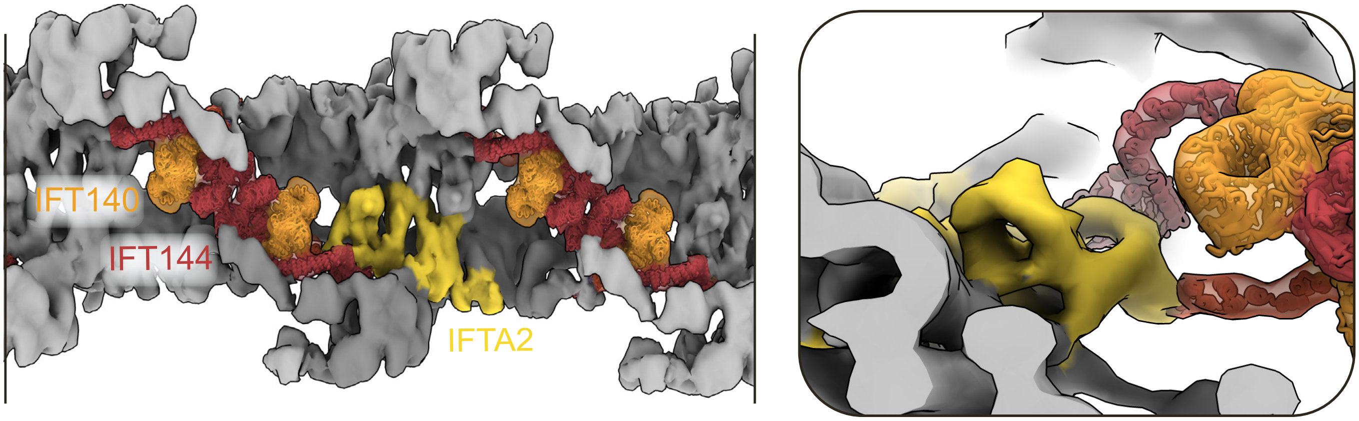

Since IFTA1 is linked to IFTA2 through IFT122, it followed that IFTA2 would reside next to our newly placed IFTA1. Indeed, we identified an additional WD domain in the adjoining density (yellow), which contained the characteristic WD/TPR domain angles of IFT121.

Step 5: Fit and adapt existing IFTA2 models into density

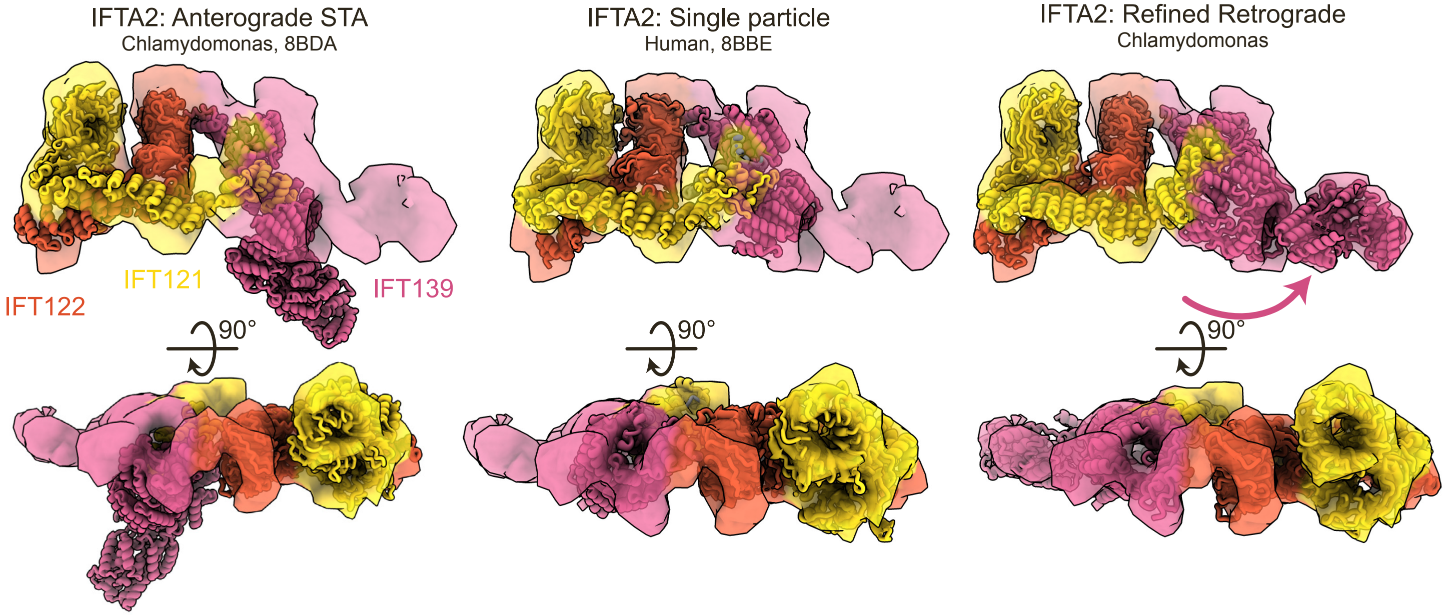

We fit in IFTA2 from our anterograde structure (left) and from single particle structures (center, 8BBE shown) into our density. They showed good agreement with the density, but we identified a conformational change to fit IFT139 into the density. We used the IFT139/121 Alphafold2 prediction 0, since it best represented the straight conformation of IFT139 in the density (final refined retrograde model, right).

Step 6: Expand model into repeating density

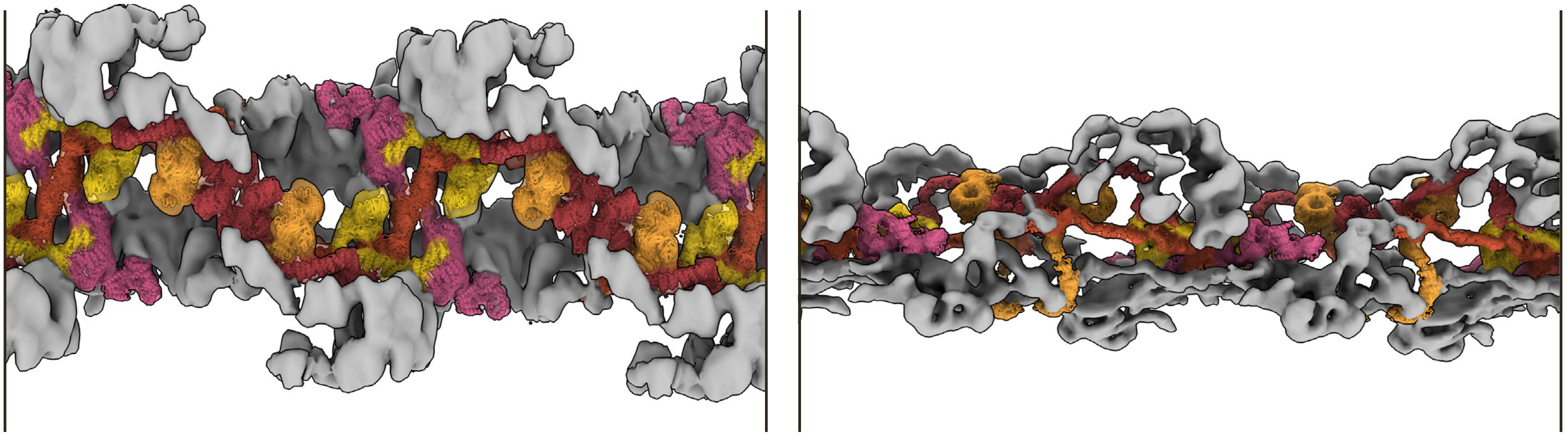

We again expanded the built region of the map into the adjacent repeats.

Observation: Similarity with PDB structures

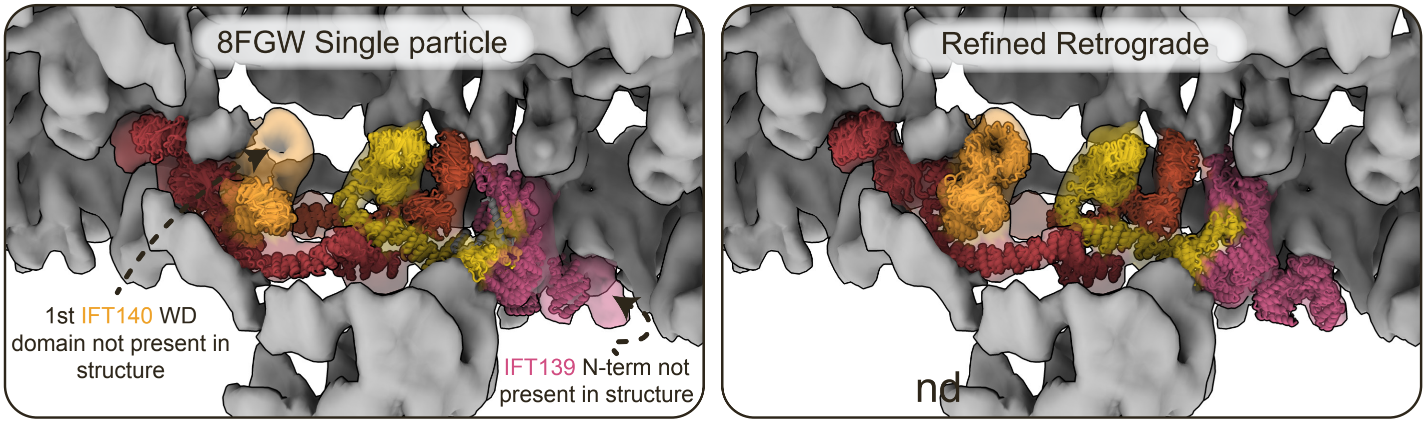

We noted that the high-resolution experimental single particle structure of human IFTA in which the bridge is present (PDB 8FGW) fits into our density (left), and corresponds well with our refined IFTA model (right)

Step 7: Identification of IFTB2

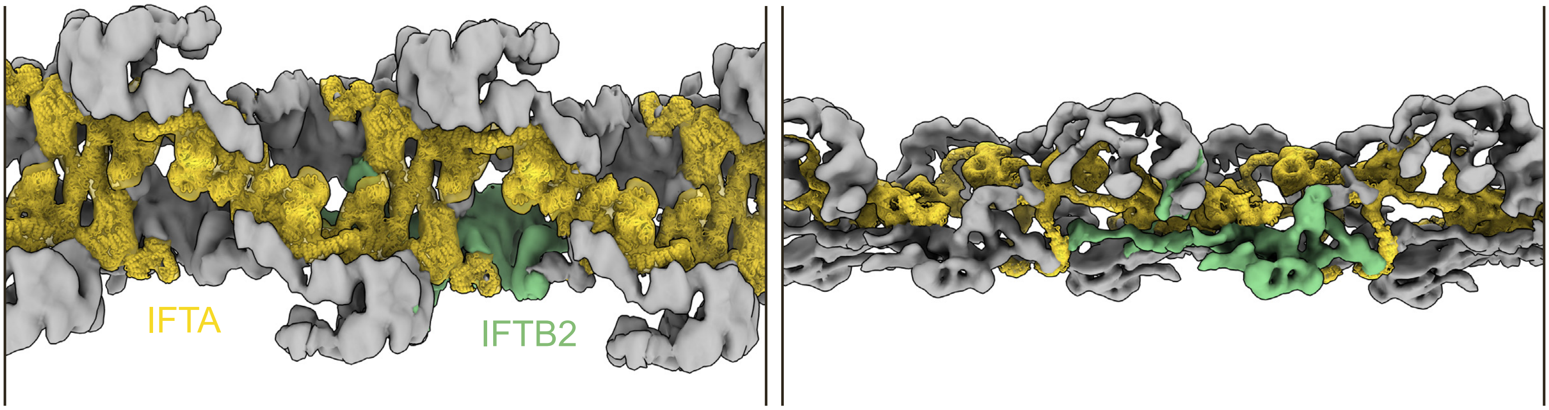

Fitting the anterograde model of IFTB2 into the remaining density provided strong matches (green region, and next step). Density covered by the refined IFTA model colored yellow.z

Step 8: Fit and adapt two copies of anterograde IFTB2 into density

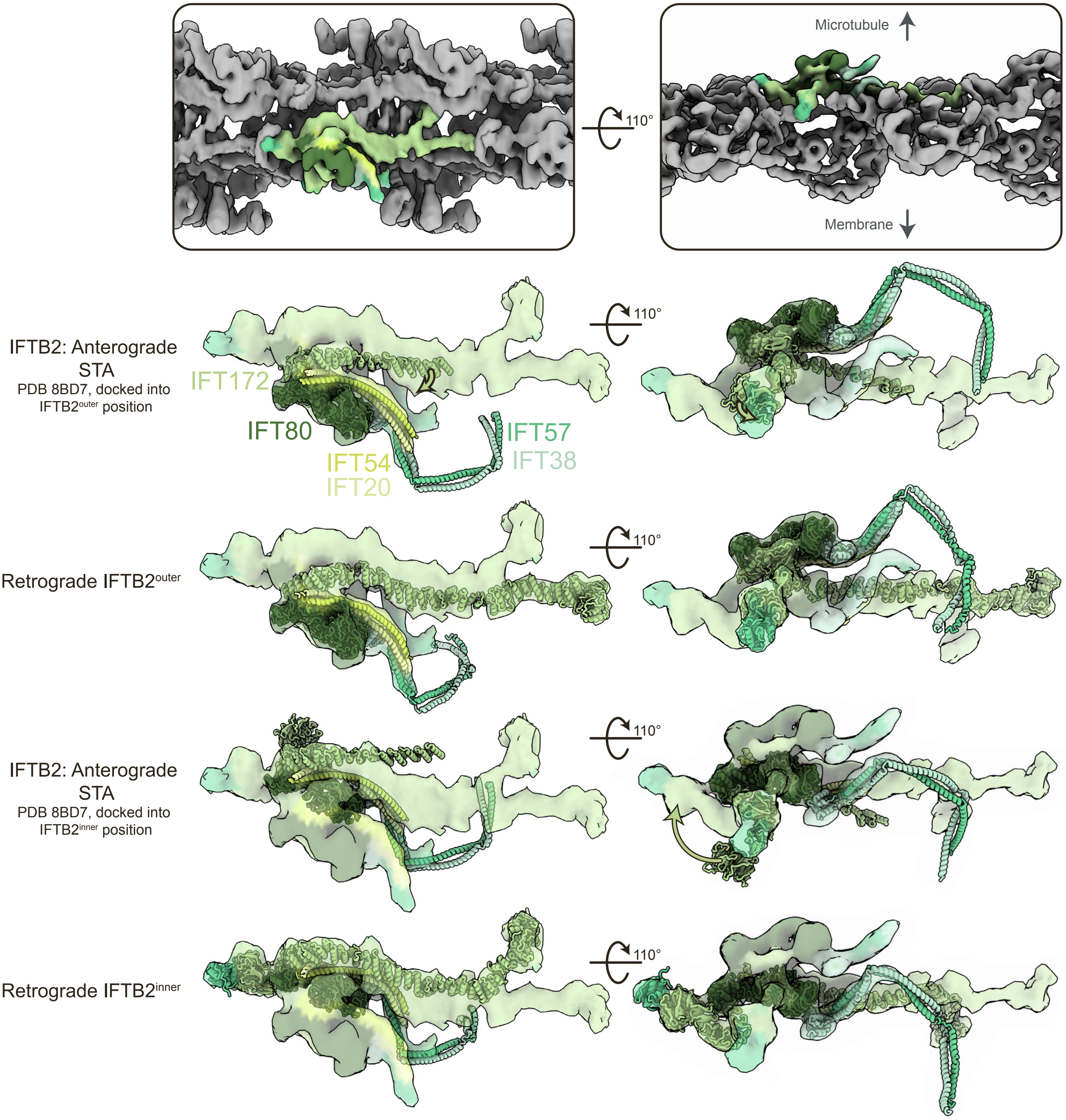

Comparisons between the density and IFTB2 models. First row, orthogonal views of the anterograde IFTB2 structure (PDB 8BD7) docked into the IFTB2^{outer} position. The model fit into this region strongly as a rigid body. Second row, orthogonal views of the refined IFTB2^{outer} model, showing the introduction of the IFT172 WD domains into the continuous density. Third row, docking of anterograde IFTB2 into the IFTB2^{inner} position shows a strong match with the remaining density, with a mismatch in the TPR domains and in the N-terminal WD domain (arrow into density). Bottom row, the refined retrograde model, showing the introduction of the TPR domain into the continuous density, and the movement of the IFT172-WD/IFT57-CH domain into the density .

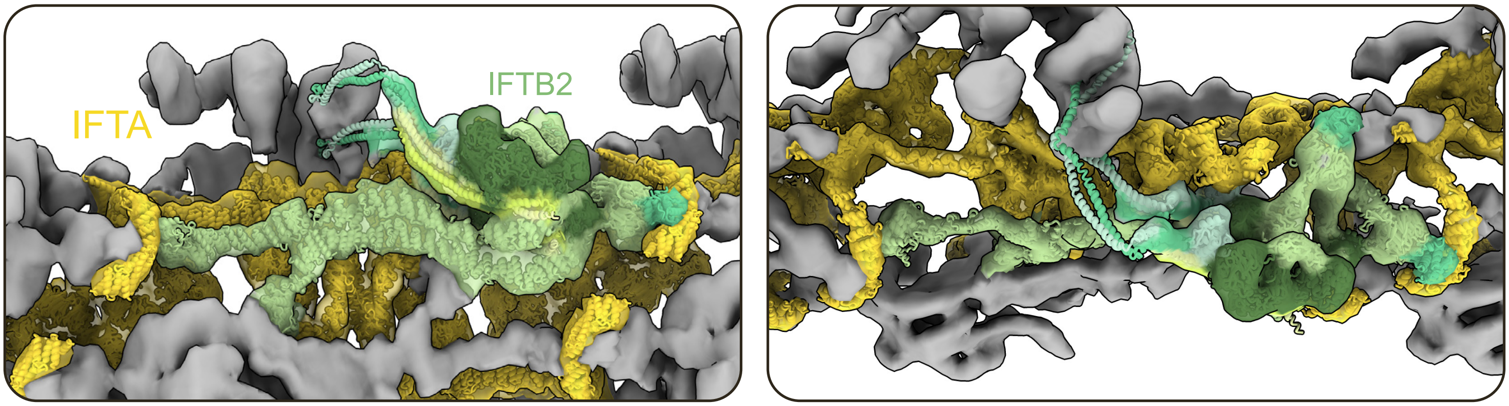

Views of the final IFTB2 model into the retrograde density.

Step 9: Fit anterograde IFTB1 core into remaining density

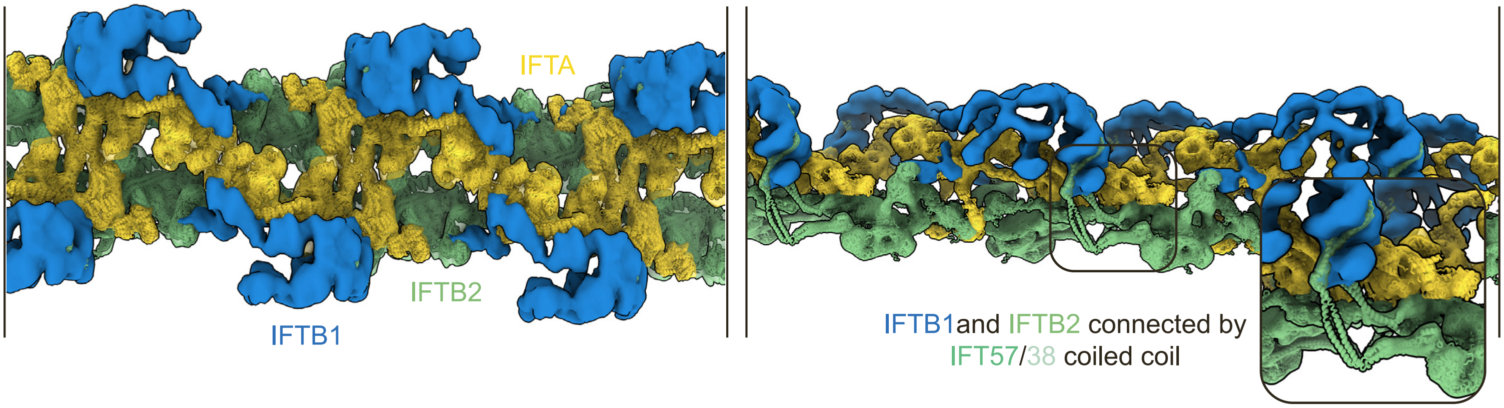

Once the IFTB2 model (green) is expanded into the adjacent repeats, the only remaining density are the blue regions. Additionally, we could infer the position of the remaining subcomplex, IFTB1, from its known link to IFTB2 through the IFT57/38 coiled coils (inset)

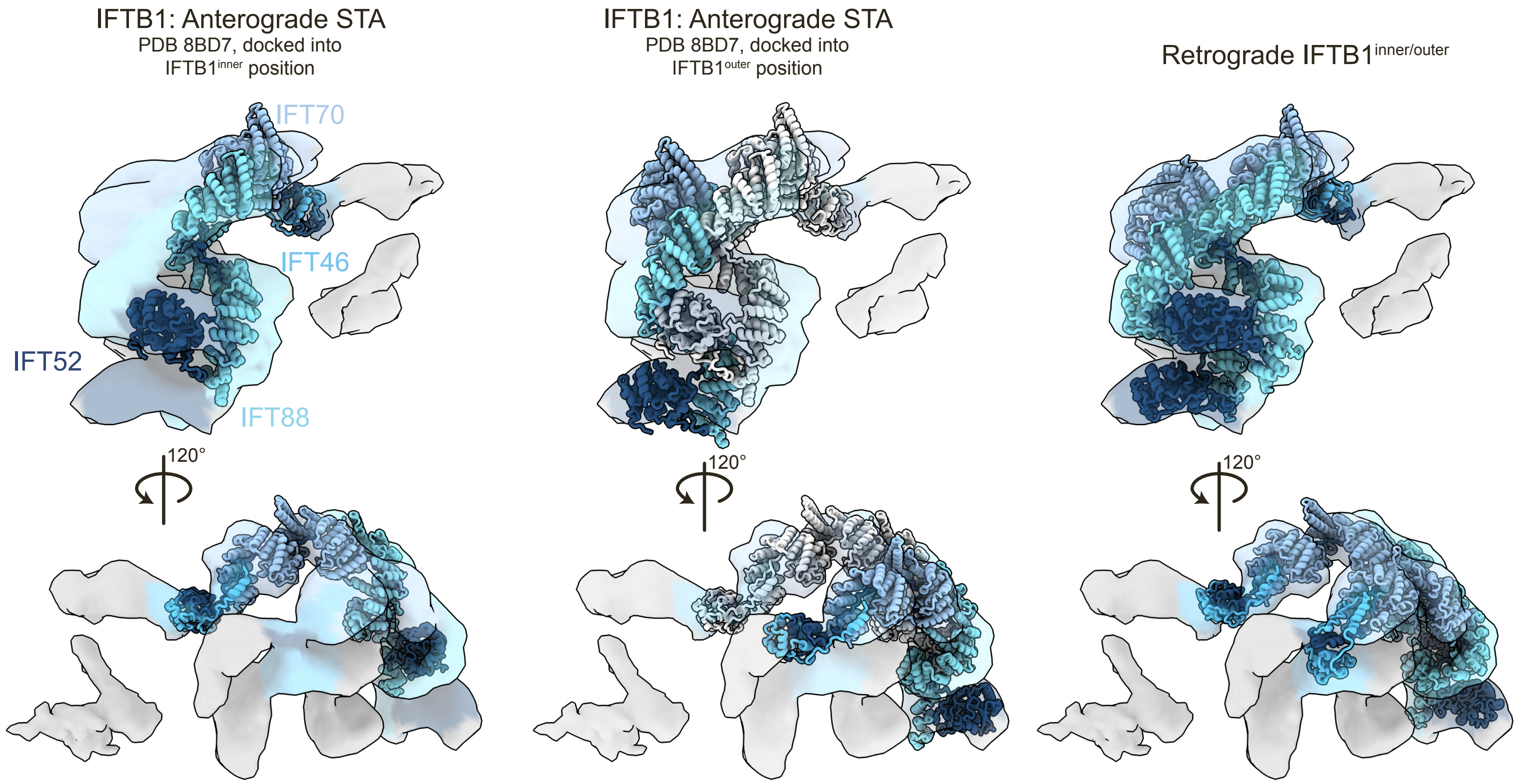

We identified a twist in the density corresponding to the TPR superhelices of IFT88 and IFT70. We docked in the anterograde IFTB1 model first into the IFTB1inner position (left), followed by the outer position (center, same docked complex in left panel is now coloured white). Right, refined IFTB1 in the retrograde model.

Step 10: Unfilled densities remains at the end of IFTB1inner

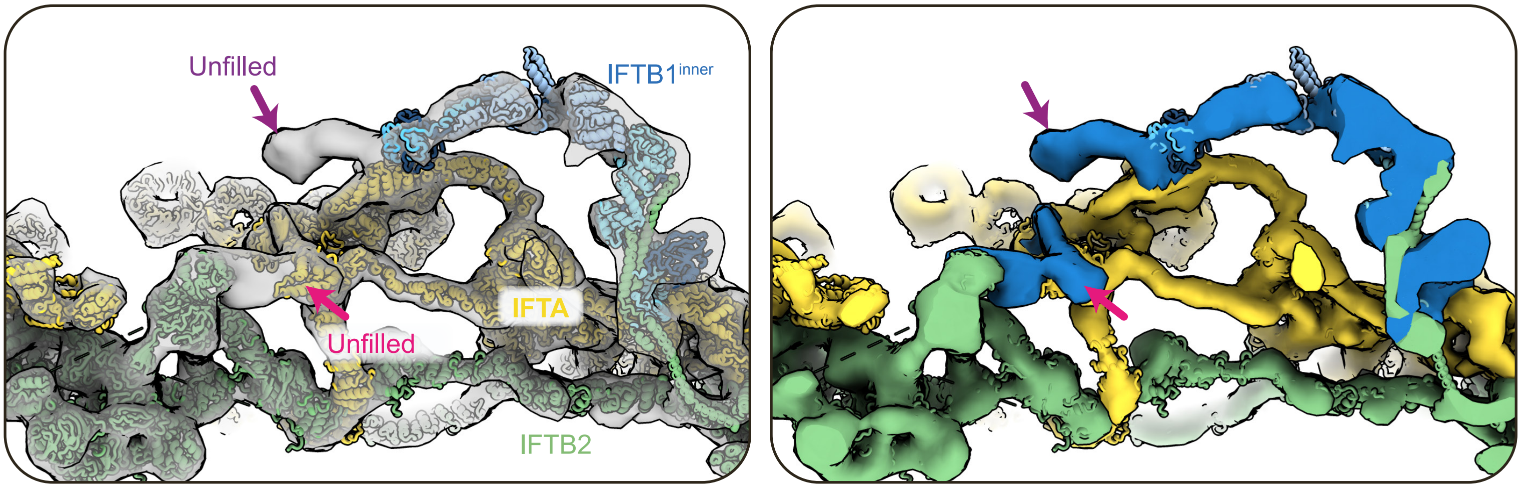

There was not a strong match in the density for IFT81/74 in the same conformation as in the anterograde train. Instead, we noted an unfilled density at the end of IFTB1inner, beyond the IFT52/46 dimer. Since this region is known to interact with IFT81/74 C-terminus61 in the "traditional" conformation, we attempted to model this interaction.

Step 11: Fit in AF models of IFT81/74/27/25/22

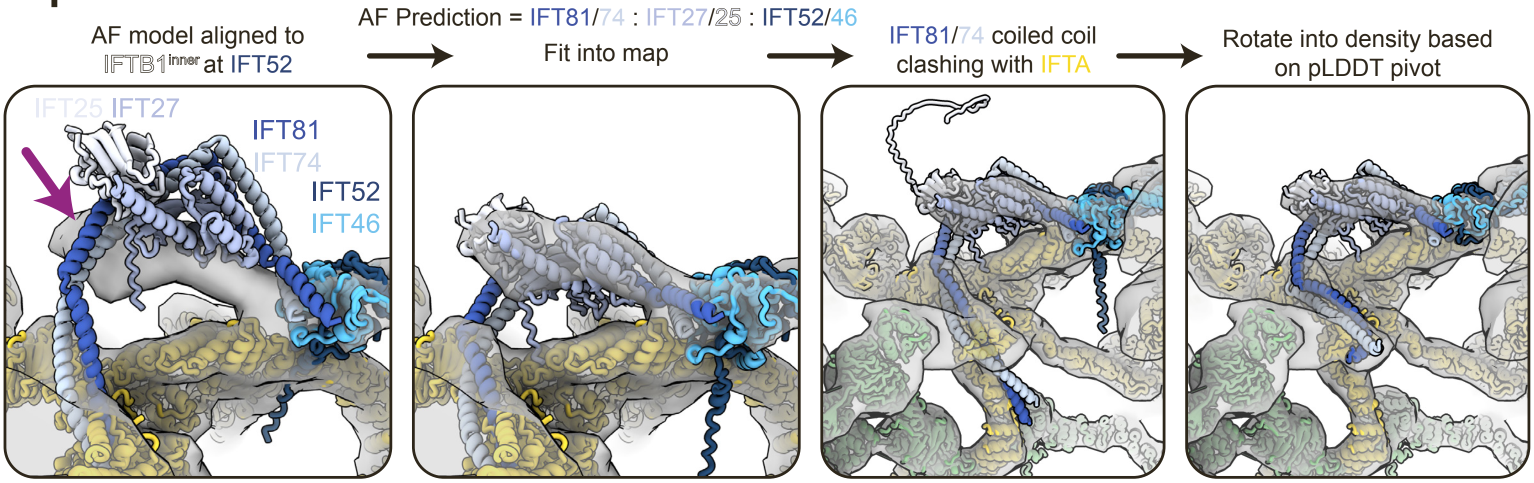

We first docked in the IFT81/74/27/25/52/46 AF prediction by aligning the IFT52 region with the same region in refined IFTB1^{inner} (Chimerax matchmaker). We next fit it into the density, achieving a strong match between the model and the density. Next, we noted a clash in the coiled coil region of IFT81/74 with IFTA (yellow), and so remodelled this region at flexible hinges identified by the pLDDT score of the Alphafold2 prediction.

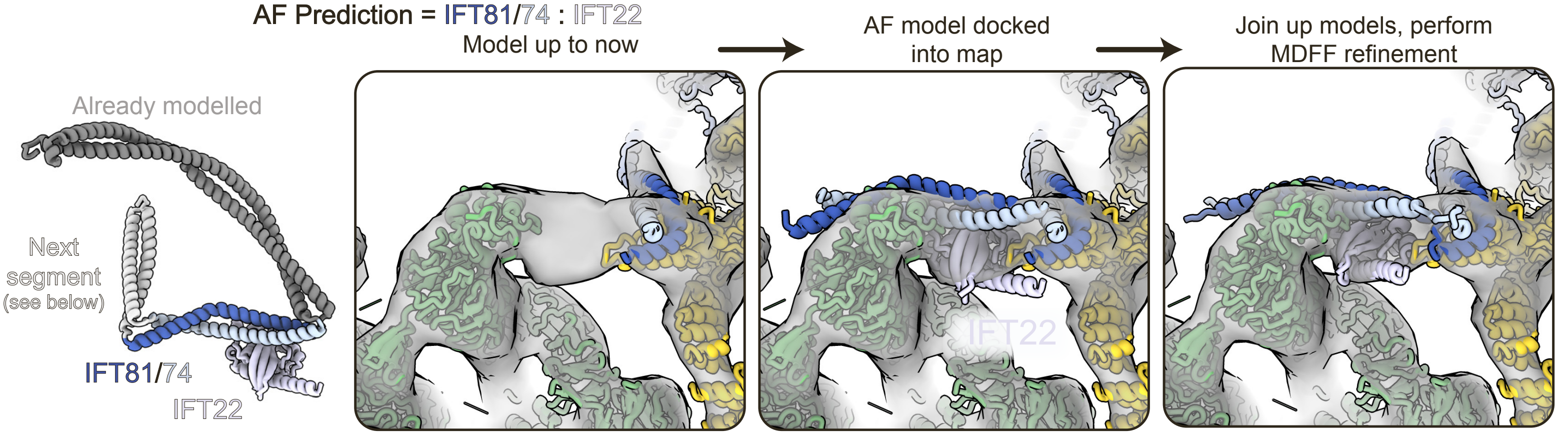

At the N-terminal end of the IFT81/74 model there was an extra unmodelled density. We fit in the preceeding coiled coil segment of IFT81/74, which contains the IFT22 binding site. This again fit the density exactly, and we joined up the AF prediction and the model and perfomed MDFF.

Step 11 continued

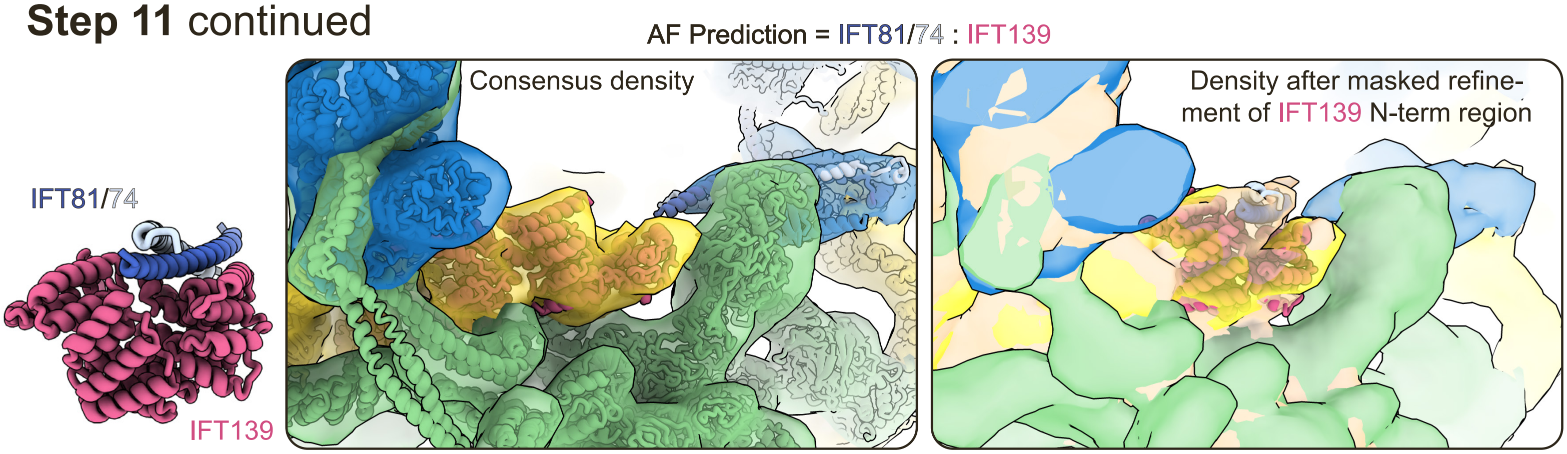

Now, the preceeding IFT81/74 segment was in close proximity to IFT139, and corresponded to the same region and orientation that we identified in the anterograde train. We performed focussed refinement of the IFT139 density here, and identified an extra density above the cleft in IFT139. This corresponded exactly with the IFT139/81/74 AF prediction, which we again fit into the density and joined with the adjacent segment.

Step 12: Low-confidence localisation of IFT81/74^{outer} into unmodelled density

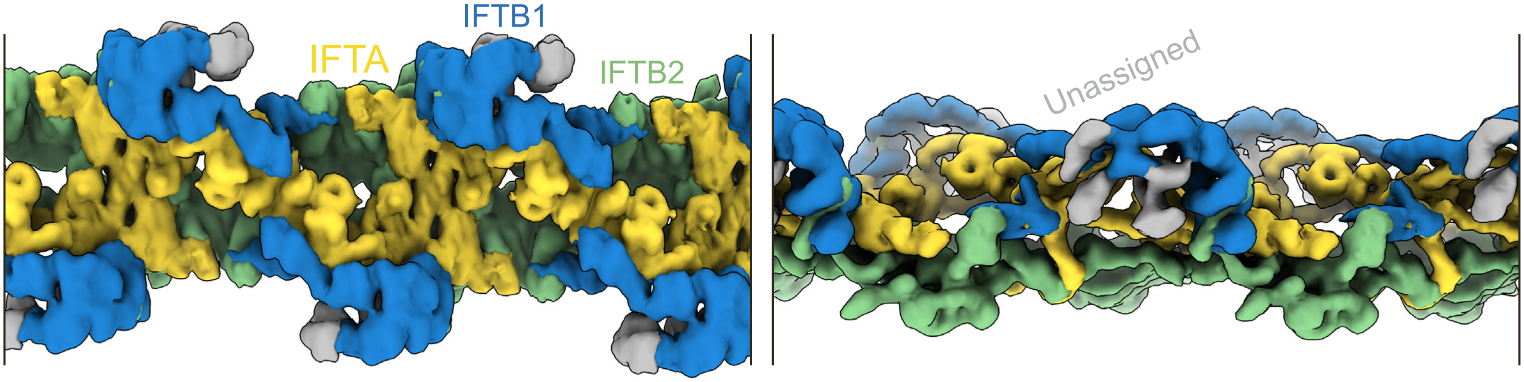

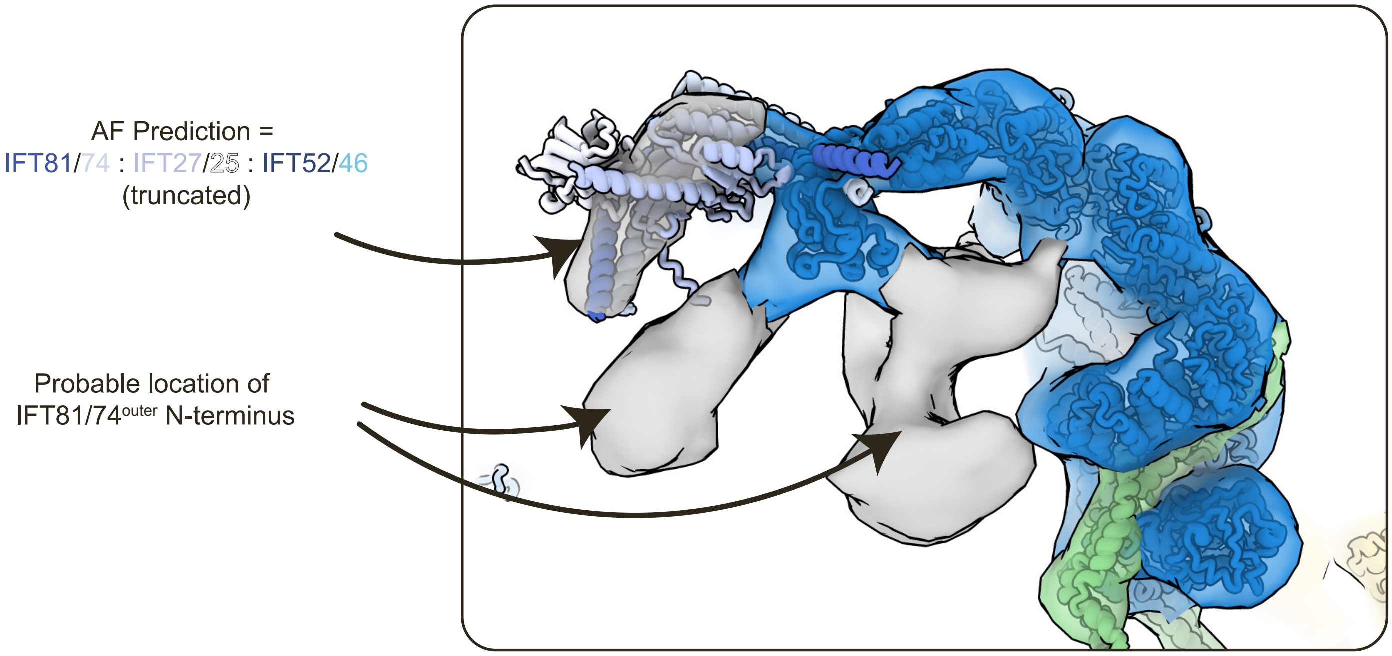

The modelled regions now cover most of our density (IFTA, yellow, IFTB1 blue, IFTB2 green, unmodelled grey). We identified a similar density that likely corresponds to the IFT81/74/27/25/52/46 block of IFTB1^{outer}, however we were unable to confidently trace the preceeding coiled coils of IFT81/74 in the remaining low-resolution density. As such, we left it unmodelled in our submitted coordinates.

Comparison between IFTB subcomplexes in anterograde and retrograde trains

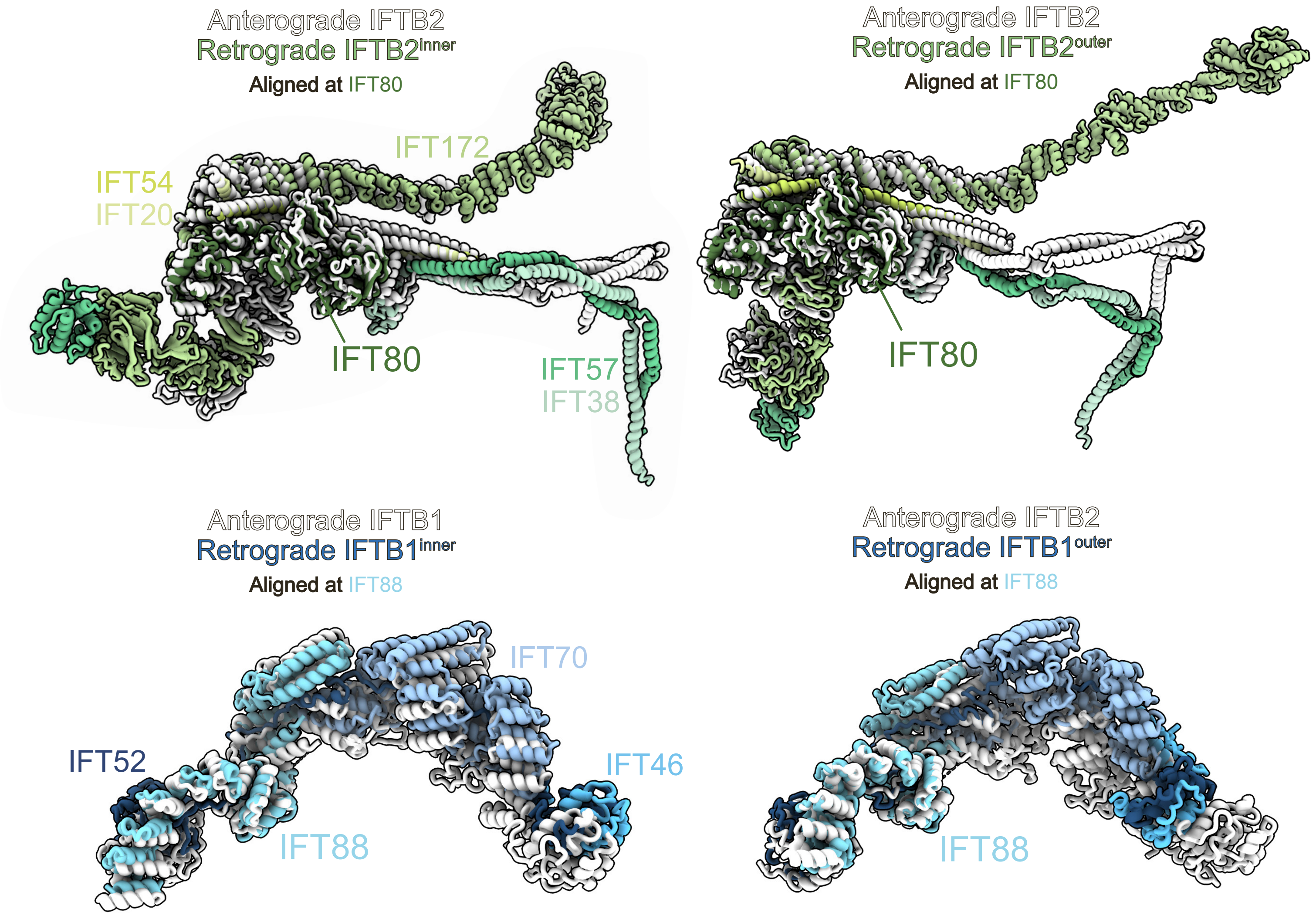

Comparisons between IFTB1 and IFTB2 in the anterograde train, and the same subcomplex in the retrograde model. Left, IFTB1/2^{inner} compared to anterograde, right, IFTB1/2^{outer} compared to anterograde. In general, only minor modifications occur in our remodelling process, with the core of the subcomplexes acting as rigid bodies.
