# Supplementary material for: Extensive structural rearrangement of intraflagellar transport trains underpins bidirectional cargo transport
Source: Cell. 2024 Aug 22;187(17):4621–4636.e18. doi: 10.1016/j.cell.2024.06.041 (PMC11349379; doi:10.1016/j.cell.2024.06.041)
Supplement: Methods S2. Assembline modeling fit library scores, related to Figure 5 [file mmc2.pdf]

Methods S2

Assembline modelling fit library scores, related to Figure 5

Assembline fitting library scores of IFTA Subcomplexes

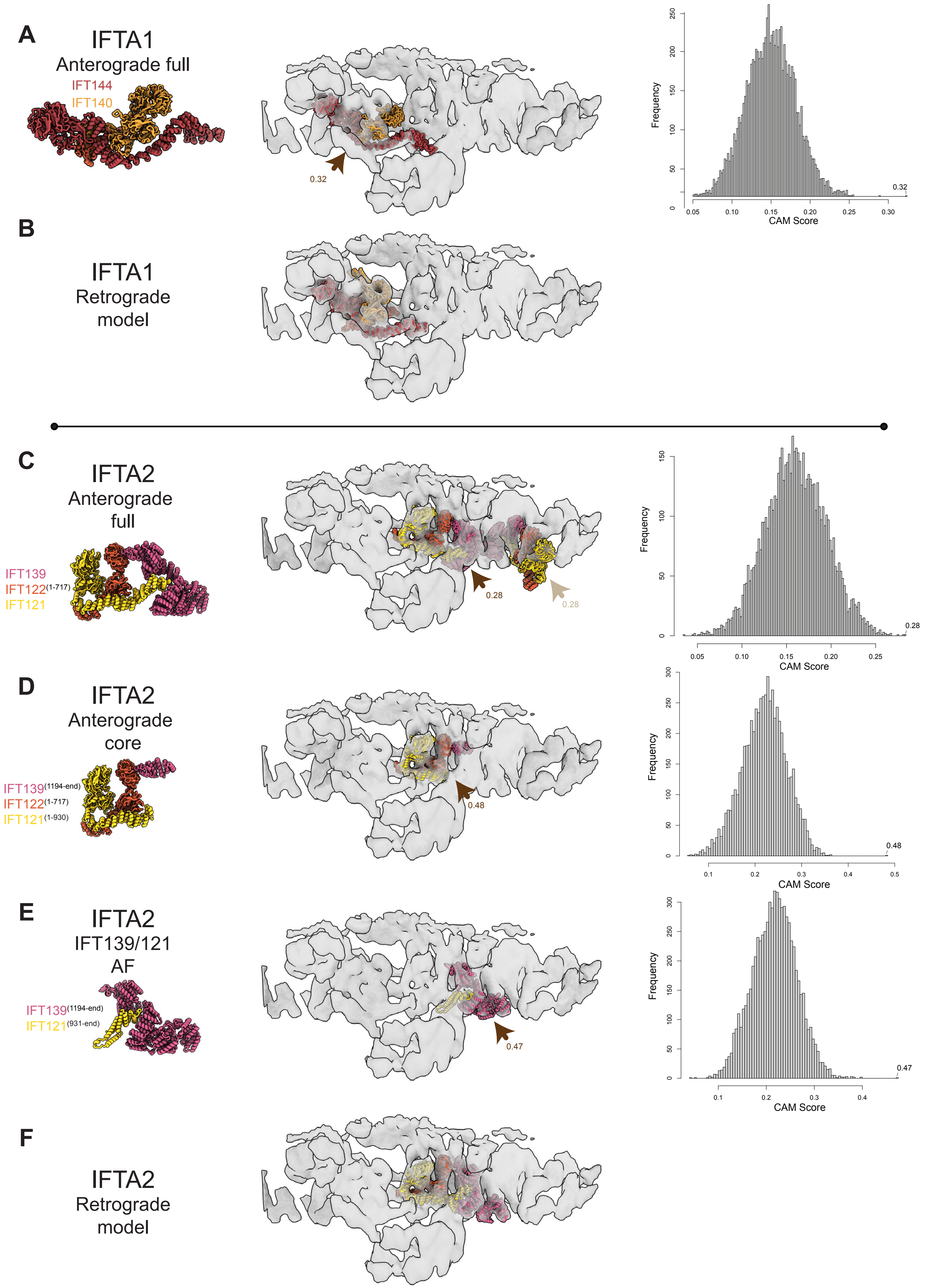

A – Results from Assembline fit library generation for the full IFTA1 complex. This process involved systematic assessment of the quality of fit of a molecular model at different positions and rotations throughout an EM density. In Chimera, the quality of the fit is calculated for each pose using the correlation about mean (CAM) metric. A higher CAM score corresponds to a stronger match between the density and the model at that pose. A histogram of the CAM score at each position shows that the fit shown is by far the strongest match of the model to the density.
B – IFTA1 in the final refined retrograde model shows that we placed it in the same position as the strongest CAM score fit.
C – Systematic fitting of the full anterograde IFTA2 complex has a strong match for our assigned retrograde position (dark brown arrow), but equally good fits for other areas of the map (light brown arrow). Right, histogram of goodness-of-fit CAM score.
D – Splitting the IFTA2 anterograde complex into two halves greatly increases the match with the density. The IFTA2 "anterograde core" complex, consisting of IFT122, IFT121 and the C-terminus of IFT139, now matches the density with a single high confidence CAM score fit. Right, histogram of goodness-of-fit CAM score.
E – The other half of the IFTA2 complex, consisting of IFT139 and the C-terminus of IFT121, also now forms a single high confidence CAM score fit. Right, histogram of goodness-of-fit CAM score.
F – Our final refined IFTA2 complex in the retrograde model, showing that the positions of each component corresponds to the unambiguous CAM score fits.

For IFTB2, next page

A – Systematic fitting the full IFTB2 complex from the anterograde structure into our retrograde density shows one isolated high-confidence CAM score fit (brown arrow). The next highest scoring position (light brown arrow) has a score consistent with other fits, indicating lower confidence of its fit. Right, histogram of goodness-of-fit CAM score.
B – Removing the potentially flexible regions of IFTB2 (the IFT172 WD domain, and the IFT172 TPR repeats further away from the core) result in two high confidence CAM score positions.
Right, histogram of goodness-of-fit CAM score.
C – Systematic fitting of the IFT172/57 Alphafold2 prediction shows two strong CAM score matches with the density. Right, histogram of goodness-of-fit CAM score.
D – Systematic fitting of the IFT172 C-terminus Alphafold2 prediction finds a number of similarly high-scoring positions in the retrograde density. While the scores are high, the fact that many different positions have the same score indicates low-confidence in the placement of this domain. Right, histogram of goodness-of-fit CAM score.
E – Systematic fitting of the IFT172/140 prediction, containing the same IFT C-terminus region as in D, now has a single high-confidence CAM score position. Right, histogram of goodness-of-fit CAM score.
F – Our final refined IFTB2 complex in the retrograde model. This shows that our assignment of the two IFTB2 complexes corresponds with the unambiguous CAM score fits in B, C and E.

Assembline fitting library scores of IFTB2 Subcomplexes

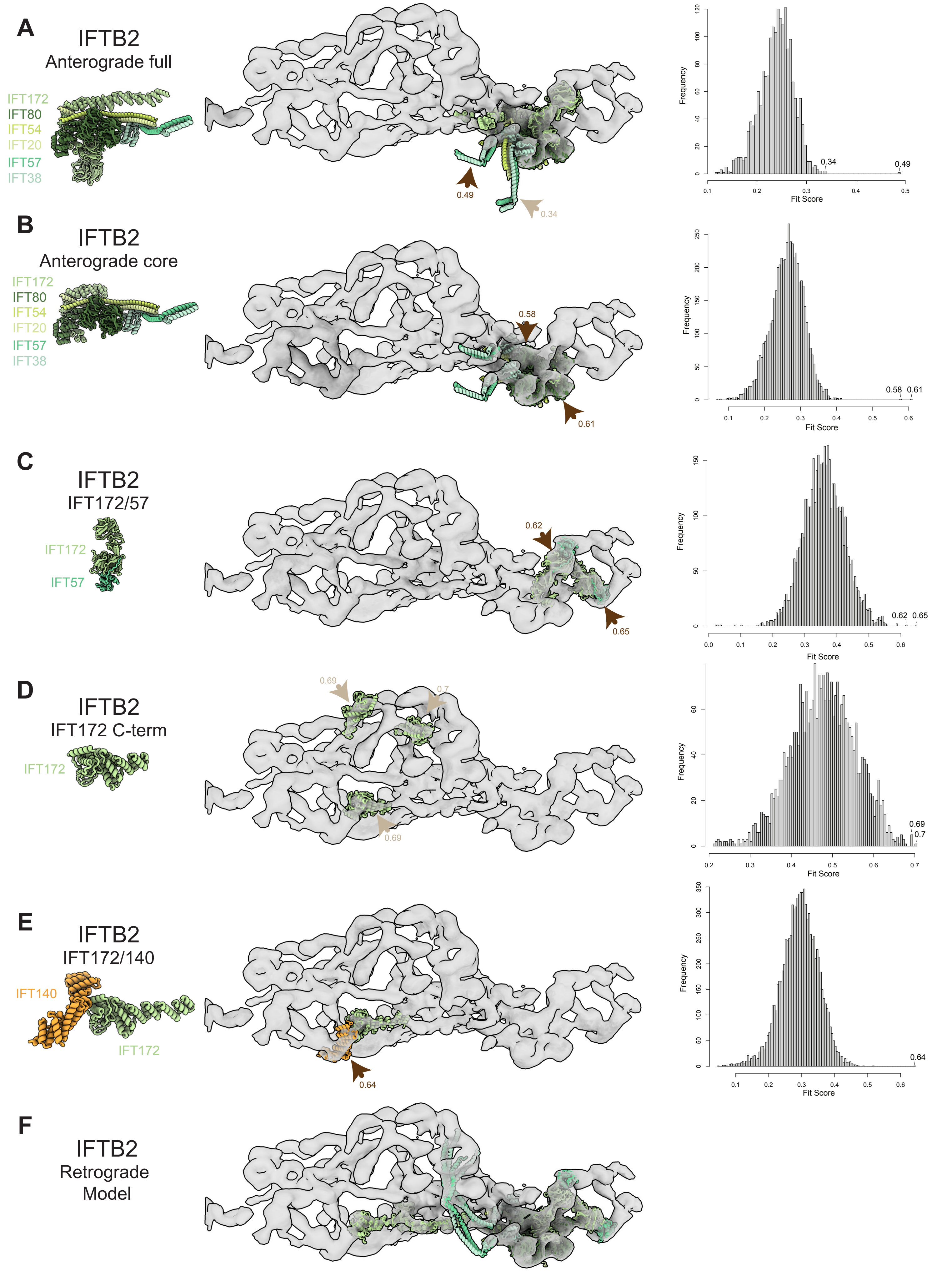

Assembline fitting library scores of IFTB1 Subcomplexes

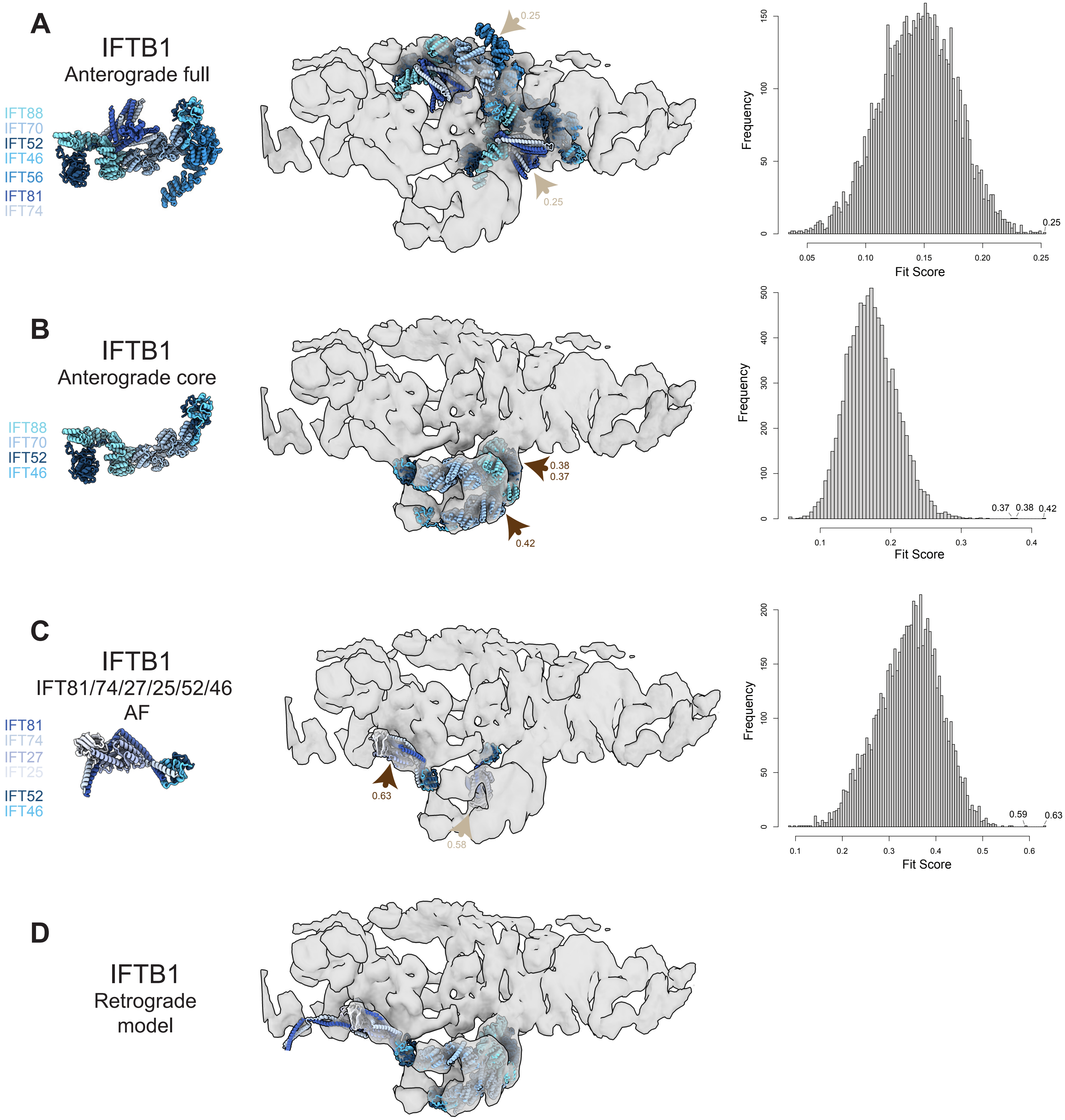

A – Systematic fitting the full IFTB1 complex from the anterograde structure into our retrograde density shows a number of similarly high-scoring positions, indicating low confidence in positional assignment. Right, histogram of goodness-of-fit CAM score.

B – Removing potentially flexible regions from the IFTB1 core complex (IFT81/74 and IFT56) now results in three high-confidence CAM score fits. Two of these positions are almost identical. Right, histogram of goodness-of-fit CAM score.

C – Systemic fitting of the IFT81/74/27/25/52/46 Alphafold2 prediction (cropped to remove some of the flexible IFT81/74 region) shows one high scoring positions (brown arrow) and other lower confidence positions (e.g. light brown arrow) that are separate from the majority of positions in the histogram. Right, histogram of goodness-of-fit CAM score.

D – Our final refined IFTB1 complex in the retrograde model. This shows that our assignment of the two IFTB1 complex corresponds with the unambiguous CAM score fits in B and C. However, consistent with C, we have only placed IFT81/74/27/25 in its higher confidence $IFTB1^{inner}$ position.
