# Supplementary material for: Extensive structural rearrangement of intraflagellar transport trains underpins bidirectional cargo transport
Source: Cell. 2024 Aug 22;187(17):4621–4636.e18. doi: 10.1016/j.cell.2024.06.041 (PMC11349379; doi:10.1016/j.cell.2024.06.041)
Supplement: Table S1. Cryo-EM data collection parameters and model statistics, related to Figure 1 [file mmc3.pdf]

Supplementary Table S1
Cryo-EM data collection parameters and model statistics, related to Figure 1.

	IFTA1	IFTA2	IFTB1(prox)	IFTB1(dist)	IFTB2
**Data Collection**					
Voltage (kV)	300				
Tilt range/Increment (°)	±60/3				
Maximum electron exposure (e-Å^{-2})	104				
Defocus range (Å)	-2 to -4				
Pixel size (Å)	3.03				
Number of tomograms	736				
**Subtomogram Averaging**					
Number of subtomograms	5896	5896	3177	3177	3891
Symmetry applied	C2 (w/ expansion)	C2	C2 (w/ expansion)	C2 (w/ expansion)	C2 (w/ expansion)
Pixel size of final map (Å)	6.06	6.06	6.06	6.06	6.06
Resolution (Å, 0.143 cutoff)	16.6	16.6	18.05	28.28	15.4
**Model Validation**					
Clashscore	18.45				
Molprobity score	2.66				
Ramachandran favour (%)	88.9				
Ramachandran outliers (%)	0.6				
